# Cyclic Tetrapeptides with Synergistic Antifungal Activity from the Fungus *Aspergillus westerdijkiae* Using LC-MS/MS-Based Molecular Networking

**DOI:** 10.3390/antibiotics11020166

**Published:** 2022-01-27

**Authors:** Junjie Han, Hanying Wang, Rui Zhang, Huanqin Dai, Baosong Chen, Tao Wang, Jingzu Sun, Wenzhao Wang, Fuhang Song, Erwei Li, Zhitang Lyu, Hongwei Liu

**Affiliations:** 1 State Key Laboratory of Mycology, Institute of Microbiology, Chinese Academy of Sciences, Beijing 100101, China; hanjj@im.ac.cn (J.H.); whanying2021@163.com (H.W.); raynaymond@outlook.com (R.Z.); daihq@im.ac.cn (H.D.); chenbs@im.ac.cn (B.C.); wangtao@im.ac.cn (T.W.); sunjz@im.ac.cn (J.S.); wangwz@im.ac.cn (W.W.); 2 School of Life Sciences, Institute of Life Science and Green Development, Hebei University, Baoding 071002, China; 3 Savaid Medical School, University of Chinese Academy of Sciences, Beijing 100049, China; 4 School of Light Industry, Beijing Technology and Business University, Beijing 100048, China; songfuhang@btbu.edu.cn; 5 Institutional Center for Shared Technologies and Facilities, Institute of Microbiology, Chinese Academy of Sciences, Beijing 100101, China; liew@im.ac.cn

**Keywords:** cyclic tetrapeptides, synergistic antifungal activity, molecular networking, *Aspergillus westerdijkiae*

## Abstract

Fungal natural products play a prominent role in the development of pharmaceuticalagents. Two new cyclic tetrapeptides (CTPs), westertide A (**1**) and B (**2**), with eight known compounds (**3**–**10**) were isolated from the fungus *Aspergillus westerdijkiae* guided by OSMAC (one strain-many compounds) and molecular networking strategies. The structures of new compounds were unambiguously determined by a combination of NMR and mass data analysis, and chemical methods. All of the isolates were evaluated for antimicrobial effects, synergistic antifungal activity, cytotoxic activity, and HDAC inhibitory activity. Compounds **1**–**2** showed synergistic antifungal activity against *Candida albicans* SC5314 with the presence of rapamycin and weak HDAC (histone deacetylase) inhibitory activity. These results indicate that molecular networking is an efficient approach for dereplication and identification of new CTPs. CTPs might be a good starting point for the development of synergistic antifungal agents.

## 1. Introduction

Fungal natural products play a prominent role in the development of pharmaceutical agents [1,2]. Cyclic tetrapeptides (CTPs) are a type of important bioactive natural product that were found to have a broad range of pharmacological properties, including antimicrobial [3,4], cytotoxic [5,6,7], and HDAC (histone deacetylase) inhibitory properties [8]. Most of the naturally occurring CTPs are obtained from fungi, such as HC toxin with cytotoxic and antimitogenic activities from *Cochliobolus carbonum* [9], apicidin with antiprotozoan activities from *Fusarium* strains [10], and microsporins A-B with antitumor activity from *Microsporum* cf. *gypseum* [11]. In recent years, some naturally occurring CTPs have been found to inhibit HDAC and regulate gene expression, which are very useful as cancer therapeutics. In addition to use as antineoplastic drugs, HDAC inhibitors (HDACis) also have anti-interstitial fibrosis [12], anti-inflammatory [13], immunomodulatory [14], and metabolic regulation activities [15].

Naturally occurring CTPs are usually produced in low yields, which limits the discovery of new CTPs. MS/MS-based molecular networking paves the way to solving this problem. As a promising strategy, molecular networking can provide guidance and improve efficiency for the discovery of new bioactive analogues with a specific skeleton from complex mixtures. In the field of bioactive peptides discovery, neoantimycin L with excellent cytotoxicity from *Streptomyces conglobatus* RJ8 [16] and thermoactinoamide A with moderate antiproliferative activity from *Thermoactinomyces vulgaris* DSM 43016 [17] were obtained based on molecular networking.

**Figure 1:**
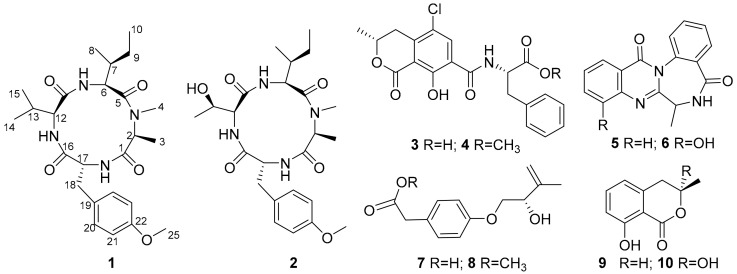
Chemical structures of **1**–**10**.

*Aspergillus westerdijkiae* is an important ochratoxin A (OTA)-producing fungus, whose genome harbors 17 non-ribosomal peptide synthetase (NRPS) genes [18]. However, most NRPS genes are unexpressed under standard laboratory conditions. In this study, we used the one strain-many compounds (OSMAC) method to activate silenced genes and MS/MS-based molecular networking to search for novel and bioactive peptides from *A. westerdijkiae* L1295. As a result, two new cyclic tetrapeptides, westertides A (**1**) and B (**2**), and eight known compounds (**3**–**10**) were obtained (Figure 1). This work describes the details of the isolation, structure elucidation, and biological activities of secondary metabolites from *A. westerdijkiae* L1295.

## 2. Results

**Figure 2:**
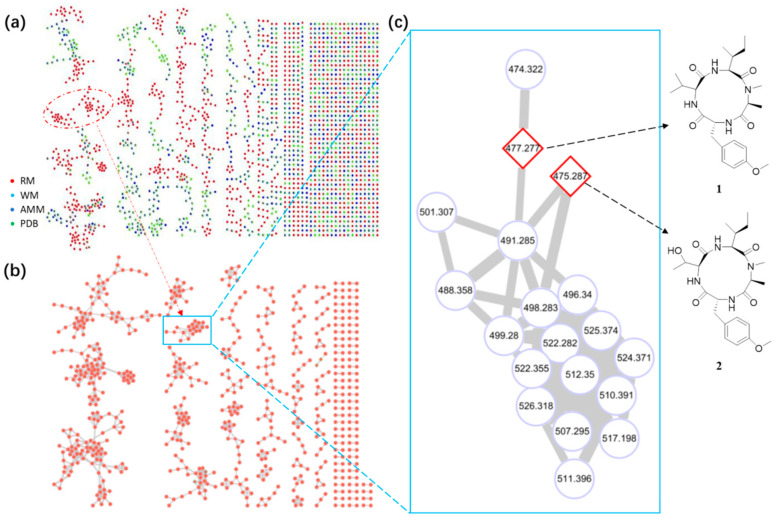
Metabolic analysis of crude extracts from *A.* *westerdijkiae* L1295. (**a**) Tandem MS/MS-based full molecular networking cluster analysis of different culture extracts of *A.* *westerdijkiae* L1295. (RM: Rice medium; WM: wheat medium; AMM: *Aspergillus* Minimal Medium; PDB: Potato-Dextrose Broth) of the fungus. (**b**) Molecular networking of *A.* *westerdijkiae* L1295 fermented on rice. (**c**) The specific subnetwork predicted to contain CTPs in the MS/MS-based molecular networking. The full GNPS network and subnetwork are presented in Figures S1 and S3 in the Supplementary Materials.

In this study, a molecular networking-OSMAC strategy was applied to accelerate the discovery of cyclic tetrapeptides. First, the fungus *A. westerdijkiae* L1295 was fermented in different culture media and conditions using the OSMAC method (Table S1). Then, the ethyl acetate extracts were further investigated by UPLC-HRMS/MS. The LC-MS/MS data were used to generate a visualized molecular networking that was further annotated by Cytoscape 3.8.2. From the full molecular network, several independent families of molecules were obviously visualized in the crude extracts of *A. westerdijkiae* L1295 fermented on rice, which were different from the other crude extracts (Figure 2 and Figure S1). Further analysis of the molecular network found that a cluster with 19 nodes represented a peptide family, showing MS/MS patterns containing the dipeptide [Ala-Phe] fragment (*m*/*z* 219.1), which has been widely found in the peptide family [19,20,21] (Figure 2 and Figure S2). Guided by MS/MS and molecular networking, two new cyclic tetrapeptides, westertides A (**1**) and B (**2**), with eight known compounds ochratoxin A (**3**) [22], ochratoxin A methyl ester (**4**) [23], circumdatin F (**5**) [24], circumdatin G (**6**) [25], stachyline B (**7**) [26], westerdijkin A (**8**) [27], mellein (**9**) [28], and 3-hydroxymellein (**10**) [25] were obtained from the solid culture on rice medium and their structure identifications are described below.

Compound **1** was isolated as a pale amorphous solid, which possessed a molecular formula of C_25_H_38_N_4_O_5_ (9 degrees of unsaturation) on the basis of HRESIMS and NMR data (Table 1). The ^1^H, ^13^C NMR, and HSQC spectra of **1** revealed the presence of 7 methyl groups including 1 *N*-methyl [*δ*_H_/*δ*_C_ 3.32 (3H, s)/30.9] and 1 *O*-methyl [*δ*_H_/*δ*_C_ 3.78 (3H, s)/55.8], 2 methylene groups [*δ*_H_/*δ*_C_ 1.32 (1H, m), 1.82 (1H, m)/25.8; 3.66 (1H, m), 3.91 (1H, m)/35.6], 1 para-disubstituted benzene [*δ*_H_/*δ*_C_ 7.05 (2H, d, *J* = 6.9 Hz)/114.8; 7.29 (2H, d, *J* = 6.9 Hz)/132.0; *δ*_C_ 132.8 and 159.5], 6 methines including 4 characteristic α-methine signals [*δ*_H_/*δ*_C_ 4.29 (1H, d, *J* =5.8 Hz)/65.3; 4.41 (1H, m)/55.8; 4.69 (1H, m)/54.7; 5.17 (1H, dd, *J* =7.0, 10.0 Hz)/55.3], 3 amide *N*-H protons (*δ*_H_ 7.38, 8.83, and 9.88), and 4 amide carbonyls (*δ*_C_ 171.2, 173.0, 174.0, and 174.1), suggesting that **1** comprised 4 amino acid residues. HMBC correlations from H_3_-3 (*δ*_H_ 1.43) to C-2 (*δ*_C_ 54.7) and C-1 (*δ*_C_ 174.0) and from H_3_-4 (*δ*_H_ 3.32) to C-2 and C-5 (*δ*_C_ 171.2) together with the ^1^H-^1^H COSY correlations of H-2-H_3_-3 led to the identification of the *N*-Me-Ala residue. The ^1^H-^1^H COSY correlations of H_3_-10-H_2_-9-H-7-H-6 and H_3_-8-H-7 together with the HMBC correlations were detected from H-6 (*δ*_H_ 5.17) to C-5 (*δ*_C_ 171.2), C-7 (*δ*_C_ 37.7), C-8 (*δ*_C_ 17.6), C-9 (*δ*_C_ 25.8), and C-11 (*δ*_C_ 173.0); from H-7 (*δ*_H_ 2.34) to C-6 (*δ*_C_ 55.3); from H_3_-8 (*δ*_H_ 1.14) to C-6, C-7, and C-9; and from H_3_-10 (*δ*_H_ 0.98) to C-7 and C-9, which confirmed the presence of the Ile moiety. Similarly, two other amino acid units Val and *O*-Me-Tyr were completely assigned.

**Figure 3:**
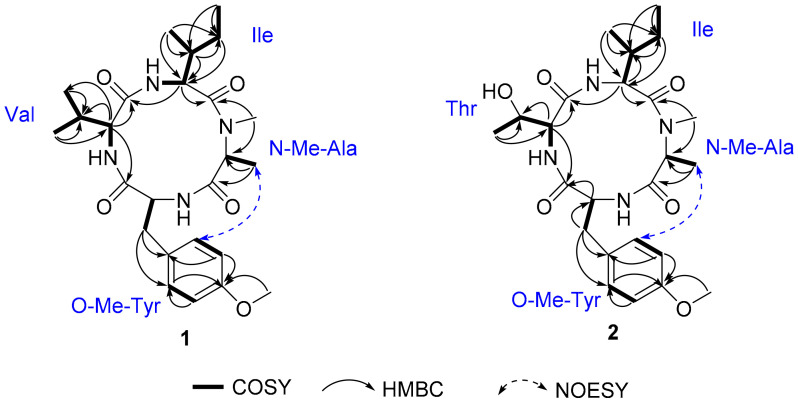
Key ^1^H-^1^H COSY, HMBC, and NOESY correlations of **1** and **2**.

**Figure 4:**
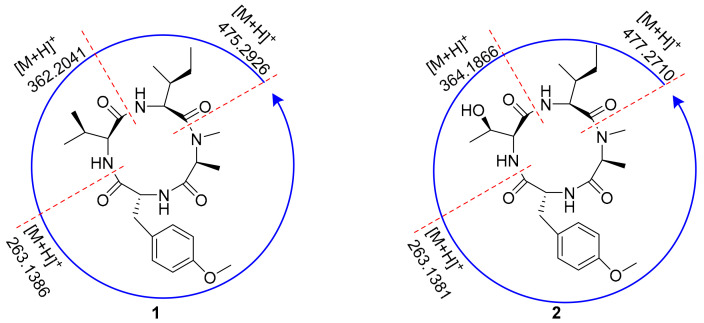
ESI-MS/MS analysis of **1** and **2**.

The amino acid sequence of **1** was deduced from the observed key HMBC correlations, NOESY correlations, and MS data. The HMBC correlations from *N*-CH_3_ (*δ*_H_ 3.32) of Ala to the Ile carbonyl group C-5 (*δ*_C_ 171.2), from H-6 (*δ*_H_ 5.17) of Ile to the Val carbonyl group C-11 (*δ*_C_ 173.0), and from H-12 (*δ*_H_ 4.31) of Val to the *O*-Me-Tyr carbonyl group C-16 (*δ*_C_ 174.1) suggested a partial sequence of *N*-Me-Ala-Ile-Val-*O*-Me-Tyr (Figure 3). The H_3_-4 (*δ*_H_ 1.43) of *N*-Me-Ala showed an NOESY correlation with H-20/24 (*δ*_H_ 7.29), indicating that **1** was a cyclic peptide, and this conclusion was also confirmed by the 9 degrees of unsaturation and the molecular formula. Additionally, the ESI-MS/MS experimental results (Figure 4 and Figure S4) also confirmed the connections of these residues as cyclo-[*N*-Me-Ala-Ile-Val-*O*-Me-Tyr].

**Figure 5:**
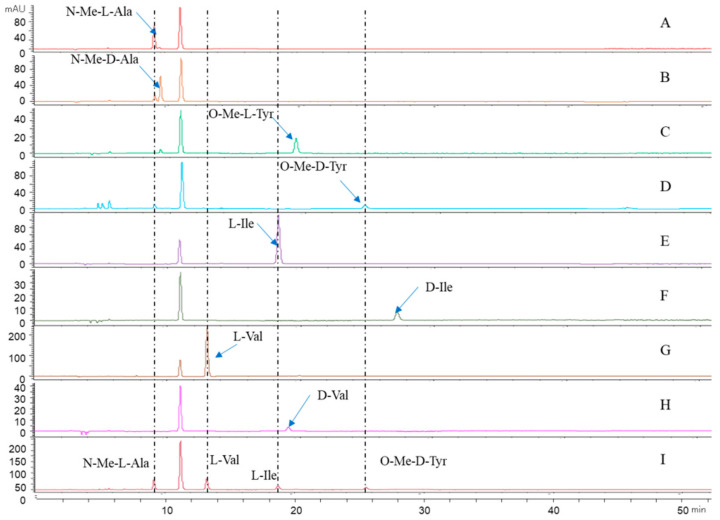
Advanced Marfey’s analysis of compound **1**. (**A**–**H**): The retention times for the FDAA derivatives of *N*-Me-l-Ala, *N*-Me-d-Ala, *O*-Me-l-Tyr, *O*-Me-d-Tyr, l-Ile, d-Ile, l-Val, and d-Val, respectively. (**I**): The FDAA derivatives of the hydrolysate of **1**. The derivatives of the acid hydrolysate and the standard amino acids were subjected to RP HPLC analysis (Kromasil C18 column; 5 μm, 4.6 mm× 250 mm; 1.0 mL/min; UV detection at 340 nm) with a linear gradient of acetonitrile (35–45%) in water (TFA, 0.01%) over 40 min.

The absolute configuration of the amino acids from compound **1** was established by the advanced Marfey’s method [29]. The mixture obtained after hydrolyzing compound **1** and further derivatization with l-FDAA was analyzed by HPLC-DAD. HPLC analyses of the mixture of hydrolysates and appropriate amino acid standards confirmed the d configurations for *O*-Me-Tyr and the l configurations for Tyr, *N*-Me-Ala, and Ile in **1** (Figure 5). Consequently, the structure of **1** was elucidated as cyclo-[l-*N*-Me-Ala-l-Ile-l-Val-d-*O*-Me-Tyr] and named westertide A.

Compound **2** was isolated as a white amorphous powder. It was assigned a molecular formula of C_24_H_36_N_4_O_6_ (9 degrees of unsaturation) based on its HRESIMS and NMR data (Table 2). The 1D NMR spectroscopic data showed that compound **2** was a cyclic tetrapeptide similar to **1** but bearing a threonine (Thr) residue with signals at *δ*_H_/*δ*_C_ 1.38 (3H, d, *J* = 6.9 Hz)/30.9 (CH_3_), *δ*_H_/*δ*_C_ 4.42 (1H, overlapped)/65.4 (CH), *δ*_H_/*δ*_C_ 4.81 (1H, m)/68.1 (CH), and *δ*_C_ 173.5 (C), instead of the valine residue. A comprehensive analysis of its relevant ^1^H-^1^H COSY, HMQC, HMBC, and NOESY correlations (Figure 3), and the ESI-MS/MS experimental results (Figure 4 and Figure S5) confirmed that **2** has the same planar structure as that of violaceomide A [20]. However, the optical rotation data of **2** ([α${]}_{D}^{25}$ = +249.5, *c* = 1.0, MeOH) were opposite to that of violaceomide A ([α${]}_{D}^{25}$ = −230.0, *c* = 0.6, MeOH), implying that they are optical isomers. The HPLC analysis of the acid hydrolysate of **2** after derivatization with l-FDAA revealed that l-*N*-Me-Ala, l-Ile, l-Thr, and d-*O*-Me-Tyr were present in **2** (Figure S6). This result shows that the main difference between compound **2** and violaceomide A is the substitution of l-*O*-Me-Tyr with d-*O*-Me-Tyr. Thus, compound **2** was assigned as cyclo-[l-*N*-Me-Ala-l-Ile-l-Thr-d-*O*-Me-Tyr] and named westertide B.

Compounds **1**–**10** showed no significant bioactivity in the antibacterial, antifungal, and cytotoxicity assays at the dose of 100 μM. In our previous work, we found that peptide-like compounds showed a synergistic antifungal effect with rapamycin [30]. So, we tested whether the new cyclic tetrapeptide compounds could also cause synergistic antifungal activity with rapamycin against *Candida albicans* SC5314. When checkerboard assays were used to obtain the MICs (minimum inhibitory concentrations) with rapamycin for achieving 90% growth inhibition, only 0.008μM of rapamycin was required together with a very low amount (6.25 μM) of compounds **1** and **2**. Based on the fractional inhibitory concentration index (FICI), westertides **1** and **2** showed effective synergism with rapamycin, and the FICI was 0.078 for both compounds **1** and **2** (Table 3). Our results showed that compounds **1** and **2** had strong synergistic antifungal activity with rapamycin. Furthermore, the effects of compounds **1** and **2** on histone deacetylation (HDAC) at the cell level were also evaluated, and compound **1** showed weak HDAC inhibitory activity, with IC_50_ of about 70 μM.

## 3. Discussion

With a low molecular weight, low hydrophobicity, and the presence of a hydrogen-bond acceptor and donor, CTPs have been demonstrated to possess diverse pharmacological activities, including antimicrobial [4], cytotoxic [5,6,7], and HDAC inhibitory bioactivities. In the last decade or so, more than 40 cyclic peptides have been approved by the FDA and EMA, such as vorinostat and romidepsin [31,32,33].

However, it is relatively difficult to discover CTPs due to their narrow distribution and low yield. As the main natural sources of CTPs, fungi have an abundance of NRPS biosynthetic gene clusters, whereas some of these genes are not expressed under normal experimental conditions. These silent gene clusters outnumber the constitutively expressed clusters by a factor of 5–10 [34]. Hence, strategies that rationally activate silent gene clusters will dramatically enhance our reservoir of potentially therapeutic small molecules [35]. In order to efficiently discover novel cyclic peptides, the molecular network and OSMAC strategy are used in combination with gene mining techniques [36]. Molecular networking can efficiently dereplicate known natural products, thus aiding the discovery of new analogues with a specific skeleton from complex mixtures [37]. The OSMAC strategy can activate some silent genes of target strains to produce more secondary metabolites and obtain novel secondary metabolites [38]. Genome mining is a powerful approach to direct the production of novel and interesting CTPs, which become relevant in the future to search for unculturable microorganisms as a new source of novel bioactive CTPs [39]. In this work, the discovery of two new cyclopeptides from *A.* *westerdijkiae* using the OSMAC strategy and the MS/MS molecular networking further expanded the structural diversity of the CTPs and the source of CTPs producers.

An estimated 1.2 billion people worldwide suffer from a fungal disease, of which 1.5 to 2 million people die of a fungal infection each year, surpassing those killed by either malaria or tuberculosis [40,41,42]. About 30% of serious infections are caused by *Candida albicans*, with a mortality rate of up to 40% [43]. Unfortunately, resistance to existing classes of drugs is on the rise due to the limited class of antifungal drugs available and the decline in new drug development. As the process of de novo antifungal discovery fails to meet clinical needs, the approach of repurposing approved drugs has drawn much attention.

Rapamycin, also called sirolimus, is characterized primarily by its antifungal activity against several human fungal pathogens, such as *Candida albicans* [44], *Cryptococcus neoformans* [45], and *Fusarium oxysporum* [46], and potent immunosuppressive activity [47]. The dual effects of rapamycin on antifungus and immunosuppression seem to effectively solve the threat of *Candida* infection when patients are treated with immunosuppressive drugs. However, rapamycin showed weak antifungal activity at the dose used to suppress the immune response in patients. The identification of synergistic actions on rapamycin against fungi can possibly solve this problem. In an early report, Tong et al. showed that some commercial or natural peptide-like compounds synergistically increased the antifungal effect of rapamycin, by targeting the Rbp1 protein (homologue of the FKBP12 protein in mammals) of *C. albicans* to increase the binding of rapamycin-Rbp1 complex with Tor1C protein [30]. In this work, we found two new natural peptide compounds, westertides A and B, showing strong synergistic antifungal activity with rapamycin from *A. westerdijkiae*. The mechanism of their synergistic antifungal effect with rapamycin may be similar to the known peptide compounds, but this requires deep investigation because they showed no antifungal effects alone.

## 4. Materials and Methods

### 4.1. General

UV data and optical rotation were recorded on a Thermo Genesys-10S UV-Vis spectrophotometer and Anton Paar MCP 200 Automatic Polarimeter, respectively. High-resolution electrospray ionization mass spectrometry (HRESIMS) data were obtained on an Agilent Accurate-Mass-Q-TOF LC/MS 6520 instrument. NMR spectral data were obtained with a Bruker AVANCE-500 spectrometer (*δ*_C_/*δ*_H_: Pyridine-*d*_5_*,* 150.4, 135.9, 123.9/8.74, 7.58, 7.22; DMSO, 39.5/2.50). Silica gel (Qingdao Haiyang Chemical Co., Ltd., Qingdao, China, 200–300 mesh), Sephadex LH-20 (GE Healthcare, Uppsala, Sweden), and ODS (50 μm, YMC CO., LTD, YMC Pack, Kyoto, Japan) were used for column chromatography. Semi-preparative HPLC was performed on an Agilent 1200 HPLC system equipped with an Agilent DAD UV−vis spectrometric detector, using a reversed-phase column (C18, 5 μm 9.4 mm × 250 mm, YMC Pack, Kyoto, Japan) with a flow rate of 2.0 mL/min. Biological reagents, chemicals, and media were purchased from standard commercial sources unless stated.

### 4.2. Fungal Material

*A*. *westerdijkiae* was isolated from the mildewed wheat, China, in September 2017. The fungus was identified mainly based on the morphological observation, molecular multilocus phylogeny analysis, and morphological features [48] (Figure S7). The fungus was deposited in China General Microbiological Culture Collection (CGMCC No. 19033).

### 4.3. Fermentation and Extraction

*A*. *westerdijkiae* was cultured on a slant of PDA at 25 °C for 5 days. To prepare inoculum, the spores of the strain on the plate were collected and adjusted to 1 × 10^6^ CFU/mL. A large-scale fermentation was carried out in 40 × 500 mL Fernbach culture flasks, with each flask containing 80 g of rice and 60 mL of distilled water (each with 0.5 mL of spore suspension), incubated at 25 °C for 3 weeks. The fermented rice substrate was extracted repeatedly with ethyl acetate by exhaustive maceration (3 × 4 L), and the organic solvent was evaporated to dryness under vacuum to afford the crude extract (20.1 g).

### 4.4. LC-MS/MS and Molecular Networking Analysis

LC-MS-MS was performed on an Agilent series 1290 Infinity HPLC instrument, coupled with a Q-TOF Mass spectrometer (Agilent Technologies Inc., Santa Clara, CA, USA), with a YMC C18 column [(YMC Co., Ltd. Kyoto, Japan) YMC-Park, ODS-A, 250 mm × 2.1 mm, S-5 μm, 12 nm, 0.5 mL/min]. The total extracts (0.5 mg/mL, 10 μL) were analyzed by LC-MS with a gradient program of MeCN−H_2_O (0.01% TFA) [0–25 min 5–80%, 25–32 min 80–100%, 32–38 min 100%; 0.5 mL/min; MS scan 150–2000 Da] and then with an automated full-dependent MS-MS scan. Mass spectral networks were assembled as described in the reference. Differentiation of the protonated molecules, adducts, and fragment ions was done by identification of the [M+H]^+^ ion. The All MS/MS data files were converted to “.mzML” format files using MSConver software and uploaded on the GNPS Web platform (http://gnps.ucsd.edu, accessed on 6 August 2021) for MN analysis using Classic mode. For the network creation, a parent mass tolerance of 0.02 Da and a fragment ion tolerance of 0.05 Da were applied. The generated molecular network was visualized in Cytoscape 3.8.2 (www.cytoscape.org, accessed on 6 August 2021) and guided the isolation of **1**–**8**. The MS/MS molecular network can be browsed and downloaded from the GNPS Web site with the following links: https://gnps.ucsd.edu/ProteoSAFe/status.jsp?task=6794bab0d59245bf875b14c6ebb84ff4 and https://gnps.ucsd.edu/ProteoSAFe/status.jsp?task=84b42a96c887412db918a18f20491b8b (accessed on 6 August 2021).

### 4.5. Isolation and Characterization Data

The EtOAc fraction was subjected to silica gel column chromatography (CC) using petroleum/ethyl acetate (P/E) in a gradient elution (*v*/*v*, 100:0, 100:1, 100:2, 100:4, 100:10) and dichloromethane/acetone (*v*/*v*, 100:0, 100:2, 100:4, 100:8, 100:12, 100:20, 0:100) to give 16 fractions (*AW*.1–*AW*.16).

Fraction *AW*.6 (0.85 g) was further separated on a silica gel column by elution with increasing concentrations of ethyl acetate in petroleum to give 15 fractions (*AW*.6-1–*AW*.6-15). Compound **9** (4.5 mg) was obtained from subfractions *AW*.6-8 (45 mg) by sephadex LH-20 chromatography eluted with dichloromethane/methanol (*v*/*v*, 1:1). *AW*.6-10 (75 mg) was purified finally by RP-HPLC with acetonitrile/water (50:50) to give **10** (13.5 mg, *t*_R_ 22.3 min).

Fraction *AW*.13 (4.3 g) eluted with CH_2_Cl_2_-Acetone (*v*/*v* 20:1) was first separated by ODS using a gradient of increasing methanol (30%, 45%, 60%, 75%, and 100%) in water to afford 25 subfractions (*AW*.13-1–*AW*.13-25). Compound **6** (30.5 mg, *t*_R_ 15.1 min) was obtained from *AW*.13-9 (152 mg) by RP-HPLC using 21% acetonitrile in acidic water (0.005% TFA). Subfractions *AW*.13-11 (170 mg) were followed by RP-HPLC using 32% acetonitrile in water to afford a mixture of **7** (9.1 mg, *t*_R_ 22.1 min), **5** (5.6 mg, *t*_R_ 31.1 min), and **8** (6.5 mg, *t*_R_ 33.5 min). Compounds **1** (2.0 mg, *t*_R_ 40.5 min) and **2** (8.0 mg, *t*_R_ 32.5 min) were obtained from *AW*.13-15 (55 mg) by RP-HPLC using 45% acetonitrile in acidic water (0.005% TFA). Compound **3** (325.0 mg) was obtained from *AW*.13-21 by recrystallization in acetonitrile. Compound **4** (20.0 mg) was obtained from subfractions and *AW*.13-22 by sephadex LH-20 chromatography eluted with methanol, respectively.

**Westertide** **A** **(1)**: pale amorphous solid; [α${]}_{D}^{25}$ +235.57 (*c* 0.5, MeOH); UV (MeOH) *λ*_max_ (log *ε*) 222 (4.03), 275 (1.30); Positive HRESIMS: *m*/*z* 475.2926 [M+H]^+^ (calcd for C_25_H_38_N_4_O_5_, 475. 2920). NMR data, see Table 1 and Figures S8–S13.

**Westertide** **B** **(2)**: pale amorphous solid, [α${]}_{D}^{25}$ +249.48 (*c* 1.0, MeOH); UV (MeOH) *λ*_max_ (log *ε*) 220 (2.78), 275 (1.43) nm; Positive HRESIMS: *m*/*z m*/*z* 477.2710 [M+H]^+^ (calcd.for C_24_H_37_N_4_O_6_, 477.2713). NMR data, see Table 2 and Figures S14–S19.

### 4.6. Absolute Configuration of Amino Acids

Compound (1.0 mg) was dissolved in 6 N HCl (2.0 mL) and heated at 110 °C for 24 h. The solutions were then evaporated to dryness and placed in a 4 mL reaction vial and treated with a 1 g/100 mL solution of FDAA (200 μL) in acetone, followed by 1.0 M NaHCO_3_ (40 μL). The reaction mixtures were heated at 45 °C for 90 min, and the reactions were quenched by the addition of HCl (1 M, 40 µL). In a similar fashion, standard *N*-Me-l-Ala, *N*-Me-d-Ala, *O*-Me-l-Tyr, *O*-Me-d-Tyr, l-Ile, d-Ile, l-Val, d-Val, l-Thr, and d-Thr, were derivatized separately. The derivatives of the acid hydrolysate and the standard amino acids were subjected to RP HPLC analysis (Kromasil C18 column; 5 μm, 4.6 mm × 250 mm; 1.0 mL/min; UV detection at 340 nm) with a linear gradient of acetonitrile (35–45%) in water (TFA, 0.01%) over 40 min. The retention times for the FDAA derivatives of *N*-Me-l-Ala, *N*-Me-d-Ala, *O*-Me-l-Tyr, *O*-Me-d-Tyr, l-Ile, d-Ile, l-Val, d-Val, l-Thr, and d-Thr were 9.1, 9.4, 19.0, 25.0, 18.1, 25.8, 12.9, 18.4, 5.2, and 6.1 min, respectively, whereas those for the FDAA derivatives of *N*-Me-Ala, *O*-Me-Tyr, Ile, and Val in the hydrolysate of **1** were 9.1 (*N*-Me-l-Ala), 25.0 (*O*-Me-d-Tyr), 18.1 (l-Ile), and 12.9 (l-Val) min, and *N*-Me-Ala, *O*-Me-Tyr, Ile, and Thr in the hydrolysate of **2** were 9.1 (*N*-Me-l-Ala), 25.0 (*O*-Me-d-Tyr), 18.1 (l-Ile), and 5.2 (l-Thr) min, respectively.

### 4.7. Evaluation of Biological Activities

#### 4.7.1. Antifungal and Synergistic Antifungal Assay

*Candida albicans* SC5314 was used as a test strain for the antifungal and synergistic antifungal bioassay. Checkerboard assays were carried out as described previously [29,30]. Overnight cultures were chosen to prepare the strain suspension with RPMI 1640 medium. RPMI 1640 was purchased from Invitrogen, and was used according to the manufacturer’s protocol, by supplementing 2% glucose, 3.45% MOS, then adjusting the pH to 7.0. Compounds were dissolved in DMSO. Final concentrations ranged from 0.002 to 2 μg/mL for rapamycin and 0.39 to 25 μg/mL for peptide-like compounds, respectively. Rapamycin was 2-fold diluted from 1 to 11 (column), while selected compounds were 2-fold diluted from A to G (row) of the 96-well microtiter plate. The fractional inhibitory concentration index (FICI) is defined as the sum of the MIC of each drug when used in combination divided by the MIC of the drug used alone. Synergism and antagonism were defined by FICI of ≤0.5 and >4, respectively.

#### 4.7.2. Cytotoxicity Assay

Cytotoxicity tests against A549, HepG2, and K562 cell lines were carried out as previously described [49]. Taxol, 5-Flourouracil, and Cisplatin were used as the positive control.

#### 4.7.3. HDAC Activity Assay

The HDAC activity of the compounds was measured using an HDAC8 Deacetylase Fluorometric (Human) Assay Kit (Cat KA4444, Abnova, Taipei, Taiwan) according to the manufacturer’s instructions. Fluorescence signal was detected with excitation at 360 nm and emission at 460 nm using a fluorescence microplate reader (Perkin-Elmer, Waltham, MA, USA). Experiments were performed in triplicate and data were analyzed using GraphPad Prism (version 6.0), Kd values were calculated by nonlinear curve fitting using a 1-site binding (hyperbola) model (Y = Bmax*X/(Kd + X).

## 5. Conclusions

Uncovered by OSMAC and molecular networking strategies, 2 new cyclic tetrapeptides (**1**–**2**), together with 7 known compounds (**3**–**10**) were isolated from *A*. *westerdijkiae*. All of the isolates were evaluated for an antifungal effect, synergistic antifungal activity, cytotoxic activity, and HDAC inhibitory activity. As a result, **1**–**10** showed no significant bioactivity in the antifungal assays and cytotoxicity assays at the dose of 100 μM. However, compounds **1**–**2** showed strong synergistic antifungal activity against *C. albicans* with rapamycin. In addtion, compound **1** showed weak HDAC inhibitory activity.

## Figures and Tables

**Table 1:** ^1^H (500 MHz) and ^13^C (125 MHz) NMR data of compound **1** in Pyridine-*d*_5_.

Pos.	1			
	*δ*_C_	*δ*_H_ (*J* in Hz)	HMBC	COSY
*N*-Me-Ala				
1	174.0 C			
2	54.7 CH	4.69 (m)	-	
3	17.1 CH_3_	1.43 (d, 4.3)	C1, C2	H-2
4	30.9 CH_3_	3.32 (s)	C2, C5	
Ile				
5	171.2 C			
6	55.3 CH	5.17(dd, 10.0, 7.0)	C5, C7, C11	H-7, 6-NH
7	37.7 CH	2.34 (m)	C6, C8, C9	H-6, H-8, H-9
8	17.6 CH_3_	1.14 overlapped	C6, C7, C9	H-7
9	25.8 CH_2_	1.82 (m), 1.32 (m)	C6, C7, C10	H-7, H-10
10	12.6 CH_3_	0.98 (t, 5.5)	C7, C9	H-9
6-NH		8.83 (d, 10.0)		H-6
Val				
11	173.0 C			
12	65.3 CH	4.29 (d, 5.8)	C11, C13, C14, C15, C16	H-13, 12-NH
13	32.6 CH	2.41 (m)	-	H-12, H-14, H-15,
14	20.3 CH_3_	1.18 (d, 4.3)	C12, C13	H-13
15	19.2 CH_3_	1.14 overlapped	C13	H-13
12-NH		7.38 (brs)		H-12
*O*-Me-Tyr				
16	174.1 C			
17	55.8 CH	4.41 (m)	-	H-18
18	35.6 CH_2_	3.66 (m), 3.91 (m)	C17, C19, C20	H-17
19	132.8 C			
20/24	132.0 CH	7.29 (d, 6.9)	C19, C21/23, C22	H-21/23
21/23	114.8 CH	7.05 (d, 6.9)	C19, C20/24, C22	H-20/24
22	159.5 C			
25	55.8 CH_3_	3.78 (s)	C22	
17-NH		9.88 (brs)		

**Table 2:** ^1^H (500 MHz) and ^13^C (125 MHz) NMR data of compound **2** in Pyridine-*d*_5_.

Pos.	2			
	*δ*_C_	*δ*_H_ (*J* in Hz)	HMBC	COSY
*N*-Me-Ala				
1	173.7			
2	54.8	4.75 (m)	C3, C4	
3	17.2	1.46 (d, 7.3)	C1, C2	H-2
4	30.9	3.38 (s)	C2, C5	
Ile				
5	171.3			
6	55.7	5.18 (m)	C5, C7, C11	H-7
7	37.6	2.40 (m)	C6, C8, C9	H-6, H-8, H-9
8	17.8	1.18 (d, 6.5)	C6, C7, C9	H-7
9	25.2	2.10 (m), 1.42 (m)	C6, C7, C10	H-7, H-10
10	12.6	0.93 (t, 7.4)	C7, C9	H-9
6-NH		9.42 (br s) ^a^		
Thr				
11	173.5			
12	65.4	4.42 overlapped	C11, C13, C14, C16	H-13, 12-NH
13	68.1	4.81 (m)	-	H-12, H-14, H-15,
14	22.3	1.38 (d, 6.4)	C12, C13	H-13
12-NH		7.38 (br s)		H-12
*O*-Me-Tyr				
16	174.0			
17	55.8	4.42 overlapped	C16	H-18
18	35.6	3.65 (m), 3.95 (m)	C17, C19, C20	H-17
19	132.8			
20/24	132.1	7.29 (d, 8.1)	C19, C21/23, C22	H-21/23
21/23	114.8	7.06 (d, 8.1)	C19, C20/24, C22	H-20/24
22	159.5			
25	55.8	3.79 (s)	C22	
17-NH		9.91 (br s) ^a^		

^a^ The position attribution of the active hydrogen refers to the data of violaceomide A [20].

**Table 3:** MIC values of compounds **1**–**2** with rapamycin against *C. abicans* SC5314.

Drugs	Anti-Fungal MICs (μM)	Synergistic Anti-Fungal MICs (μM)	FICI ^a^	Definition ^b^
Rapamycin	0.5	**-**	**-**	**-**
**1**	>100	6.25	<0.094	S
**2**	>100	6.25	<0.094	S
Amphotericin B	0.5	0.125	1.25	NS

^a^ The concentration of rapamycin in the synergy antifungal screening experiment was 0.008 μM, at which rapamycin does not show antifungal activity. As MIC alone for compounds **1** and **2** > 100 μM, we used 100 to calculate FICI, and the start concentration was 25 μM in the checkerboard assay, and the possible minimal FICI was shown. ^b^ S: synergism; NS: no synergism.

## Data Availability

Not applicable.

## Supplementary Materials

The following are available online at https://www.mdpi.com/article/10.3390/antibiotics11020166/s1, Table S1: Culture media with different compositions and conditions for *A. westerdijkiae*; Figure S1: The molecular network obtained by combining the LC-MS/MS analyses of rice fermentation extract extracts from *A.* *westerdijkiae* L1295; Figure S2: Cyclictetrapeptides-cluster and the MS/MS spectrum of each node; Figure S3: The cluster corresponding to compounds observed in the molecular networking; Figure S4: The ESI-MS/MS spectrum of **1**; Figure S5: The ESI-MS/MS spectrum of **2**; Figure S6: Advanced Marfey’s analysis of compound **2**. Figure S7: Phylogenetic analysis and morphological characters of *A.* *westerdijkiae* L1295; Figure S8: ^1^H NMR spectrum of westertide A (**1**) in pyridine-*d*_5_ (500 MHz); Figure S9: ^13^C NMR spectrum of westertide A (**1**) in pyridine-*d*_5_ (125 MHz; Figure S10: ^1^H-^1^H COSY spectrum of westertide A (**1**) in pyridine-*d*_5_; Figure S11: HSQC spectrum of westertide A (**1**) in pyridine-*d*_5_; Figure S12: HMBC spectrum of westertide A (**1**) in pyridine-*d*_5_; Figure S13: ROESY spectrum of westertide A (**1**) in pyridine-*d*_5_; Figure S14: ^1^H NMR spectrum of westertide B (**2**) in pyridine-*d*_5_ (500 MHz); Figure S15: ^13^C NMR spectrum of westertide B (**2**) in pyridine-*d*_5_ (125 MHz); Figure S16: ^1^H-^1^H COSY spectrum of westertide B (**2**) in pyridine-*d*_5_; Figure S17: HSQC spectrum of westertide B (**2**) in pyridine-*d*_5_; Figure S18: HMBC spectrum of westertide B (**2**) in pyridine-*d*_5_; Figure S19: ROESY spectrum of westertide B (**2**) in pyridine-*d*_5_.
