# Supplementary material for: Cyclic Tetrapeptides with Synergistic Antifungal Activity from the Fungus Aspergillus westerdijkiae Using LC-MS/MS-Based Molecular Networking
Source: Antibiotics (Basel). 2022 Jan 27;11(2):166. doi: 10.3390/antibiotics11020166 (PMC8868193; doi:10.3390/antibiotics11020166)
Supplement: Supplementary file 1 [file antibiotics-11-00166-s001.zip › antibiotics-1543862-supplementary.pdf]

Supplementary Materials

Cyclic Tetrapeptides with Synergistic Antifungal Activity from the Fungus *Aspergillus westerdijkiae* Using LC-MS/MS-Based Molecular Networking

Junjie Han [1,†], Hanying Wang [1,2,†], Rui Zhang [1], Huanqin Dai [1,3], Baosong Chen [1], Tao Wang [1], Jingzu Sun [1], Wenzhao Wang [1], Fuhang Song [4], Erwei Li [5], Zhitang Lyu [2,*] and Hongwei Liu [1,3,*]

1 State Key Laboratory of Mycology, Institute of Microbiology, Chinese Academy of Sciences, Beijing 100101, China; hanjj@im.ac.cn (J.H.); whanying2021@163.com (H.W.); raynaymond@outlook.com (R.Z.); daihq@im.ac.cn (H.D.); chenbs@im.ac.cn (B.C.); wangtao@im.ac.cn (T.W.); sunjz@im.ac.cn (J.S.); wangwz@im.ac.cn (W.W.)
2 School of Life Sciences, Institute of Life Science and Green Development, Hebei University, Baoding 071002, China
3 Savaid Medical School, University of Chinese Academy of Sciences, Beijing 100049, China
4 School of Light Industry, Beijing Technology and Business University, Beijing 100048, China; songfuhang@btbu.edu.cn
5 Institutional Center for Shared Technologies and Facilities, Institute of Microbiology, Chinese Academy of Sciences, Beijing 100101, China; liew@im.ac.cn
* Correspondence: lzt325@sina.com (Z.L.); liuhw@im.ac.cn (H.L.); Tel.: +86-10-64806074 (H.L.)
† These authors contributed equally to this work.

Contents

**Table S1**	Culture media with different compositions and conditions for *A. westerdijkiae*.	Page S3
**Figure S1**	The molecular network obtained by combining the LC-MS/MS analyses of rice fermentation extract extracts from *A. westerdijkiae* L1295	Page S4
**Figure S2**	Cyclic tetrapeptides -cluster and the MS/MS spectrum of each node	Page S5-7
**Figure S3**	The cluster corresponding to compounds observed in the molecular networking	Page S8
**Figure S4**	The ESI-MS/MS spectrum of **1**	Page S9
**Figure S5**	The ESI-MS/MS spectrum of **2**	Page S10
**Figure S6**	Advanced Marfey's analysis of compound **2.**	Page S11
**Figure S7**	Phylogenetic analysis and morphological characters of *A. westerdijkiae* L1295	Page S12
**Figure S8**	^{1}H NMR spectrum of westertide A (**1**) in pyridine-d_5 (500 MHz)	Page S13
**Figure S9**	^{13}C NMR spectrum of westertide A (**1**) in pyridine-d_5 (125 MHz)	Page S14
**Figure S10**	^{1}H-^{1}H COSY spectrum of westertide A (**1**) in pyridine-d_5	Page S15
**Figure S11**	HSQC spectrum of westertide A (**1**) in pyridine-d_5	Page S16
**Figure S12**	HMBC spectrum of westertide A (**1**) in pyridine-d_5	Page S17
**Figure S13**	ROESY spectrum of westertide A (**1**) in pyridine-d_5	Page S18
**Figure S14**	^{1}H NMR spectrum of westertide B (**2**) in pyridine-d_5 (500 MHz)	Page S19
**Figure S15**	^{13}C NMR spectrum of westertide B (**2**) in pyridine-d_5 (125 MHz)	Page S20
**Figure S16**	^{1}H-^{1}H COSY spectrum of westertide B (**2**) in pyridine-d_5	Page S21
**Figure S17**	HSQC spectrum of westertide B (**2**) in pyridine-d_5	Page S22
**Figure S18**	HMBC spectrum of westertide B (**2**) in pyridine-d_5	Page S23
**Figure S19**	ROESY spectrum of westertide B (**2**) in pyridine-d_5	Page S24

Table S1. Culture media with different compositions and conditions for *A. westerdijkiae*.

		Medium compositions	Conditions	
1	Rice medium	80 g rice/60 mL distilled wate	25°C	3 weeks
2	wheat medium	80 g wheat/60 mL distilled wate	25°C	3 weeks
3	Aspergillus Minimal Medium (AMM)	$MgSO_4$ 0.24g; $NaNO_3$ 6g; KCl 0.52g; KH_2PO_4 0.815; K_2HPO_4 1.05g; $FeSO_4{\cdot}7H_2O$ 5mg; EDTA 0.05g; $ZnSO_4{\cdot}7H_2O$ 22mg; H_3BO_3 11mg; $MnCl_2{\cdot}7H_2O$ 5mg; $CuSO_4{\cdot}5H_2O$ 1.6mg; $(NH_4)_6MO_7O_{24}{\cdot}7H_2O$ 1.1mg; Dextrose 1g	25°C 180 rpm	2 weeks
4	PDB	Potato Dextrose Broth 24 g in 1L distilled water	25°C 180 rpm	2 weeks

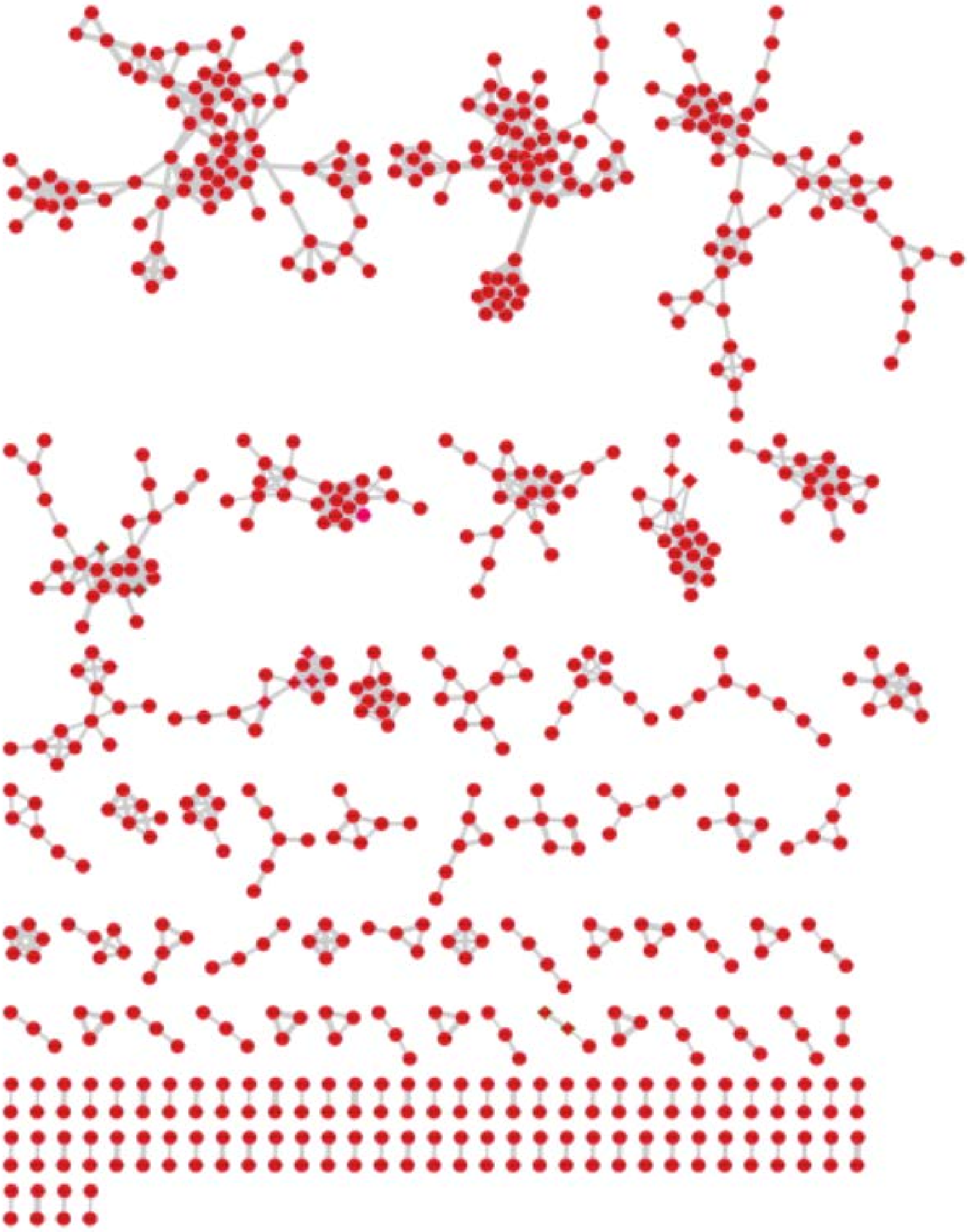

Figure S1. The molecular network obtained by combining the LC-MS/MS analyses of rice fermentation extract extracts from *A. Westerdijkiae* L1295

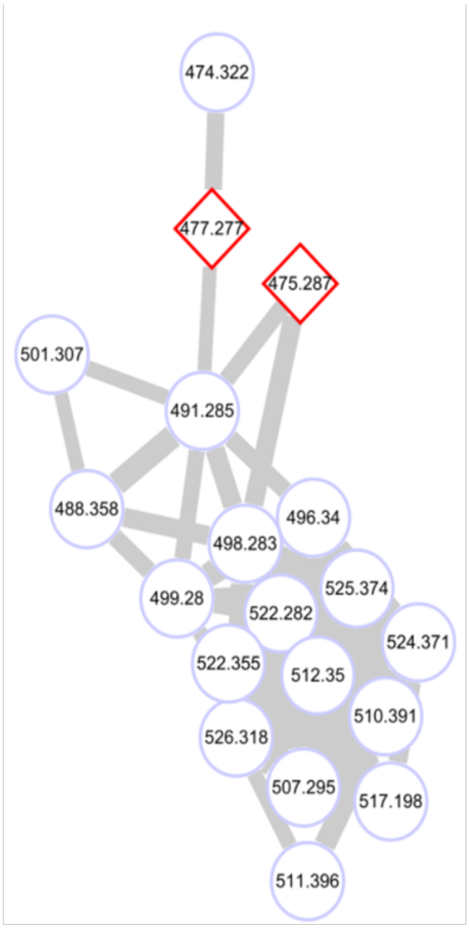
474.322
477.277
475.287
501.307
491.285
488.358
496.34
498.283
525.374
499.28
522.282
524.371
522.355
512.35
510.391
526.318
507.295
517.198
511.396

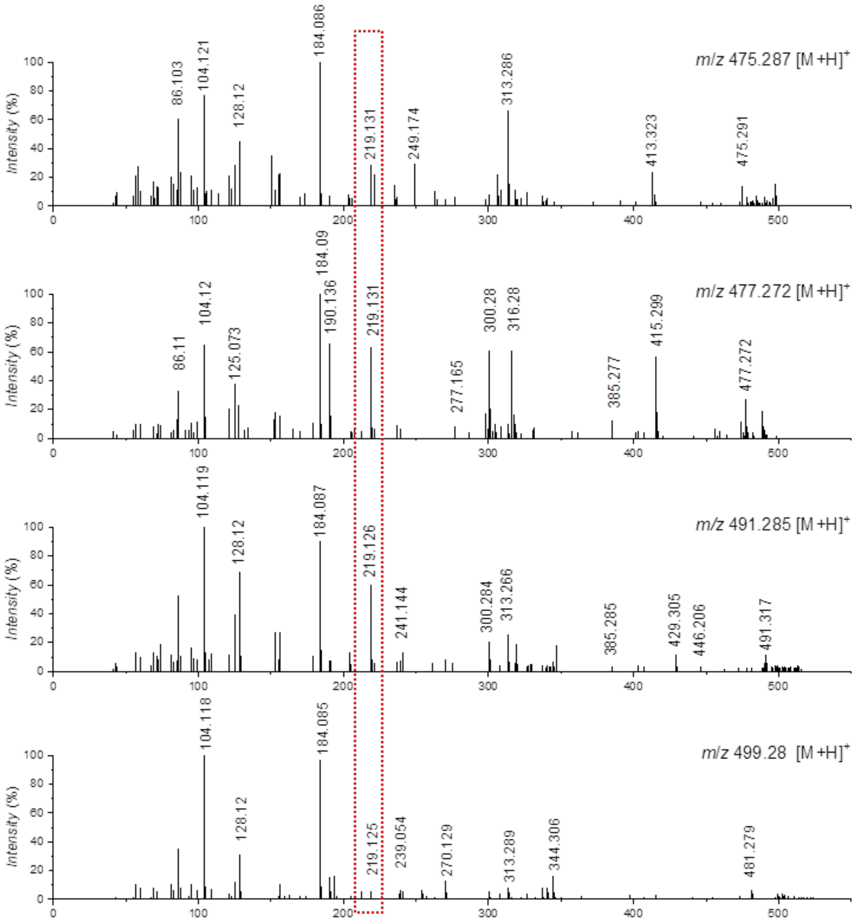
m/z 475.287 [M+H]+
m/z 477.272 [M+H]+
m/z 491.285 [M+H]+
m/z 499.28 [M+H]+
Intensity (%)

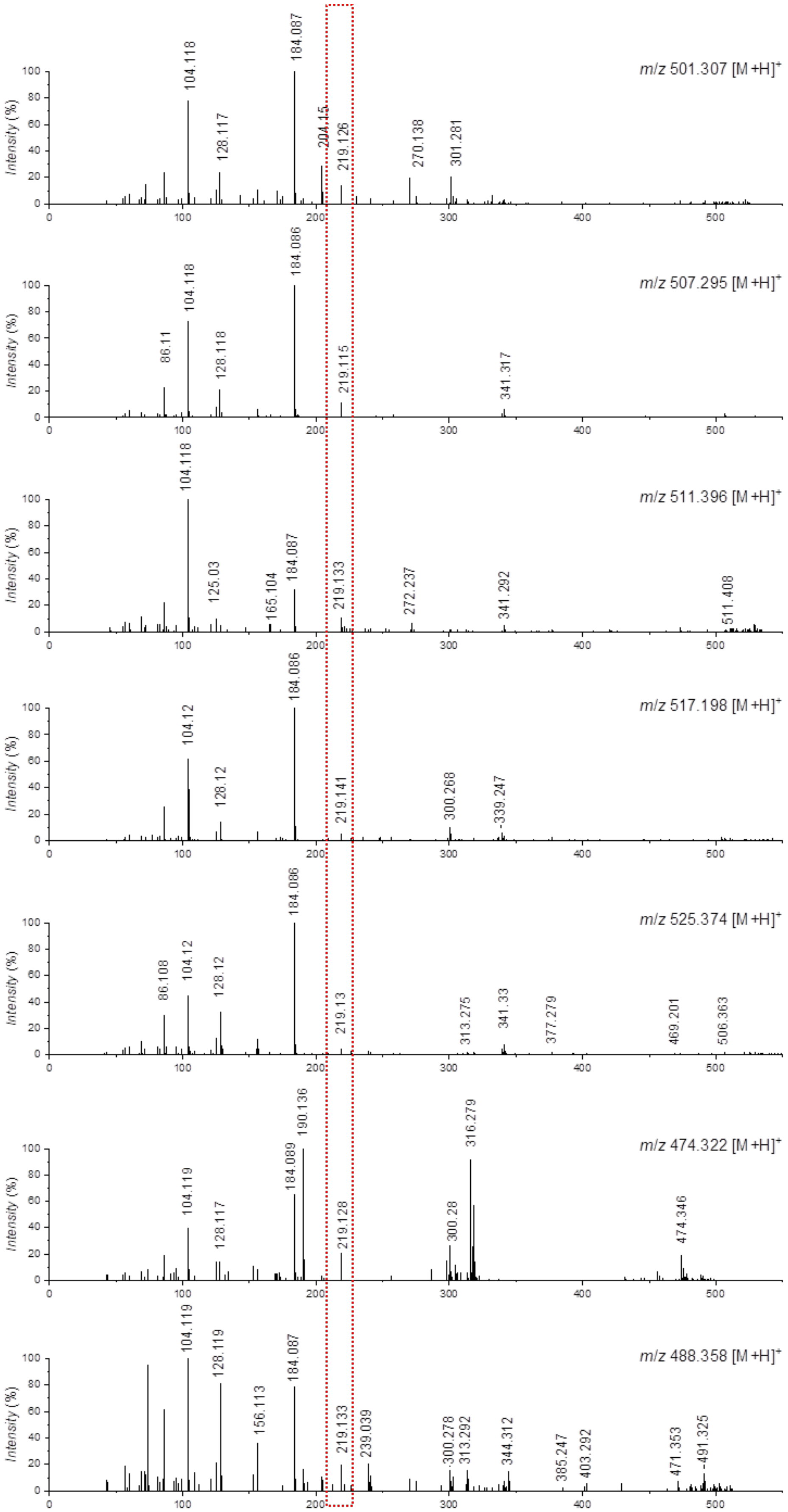

m/z 501.307 [M+H]+
m/z 507.295 [M+H]+
m/z 511.396 [M+H]+
m/z 517.198 [M+H]+
m/z 525.374 [M+H]+
m/z 474.322 [M+H]+
m/z 488.358 [M+H]+
Intensity (%)

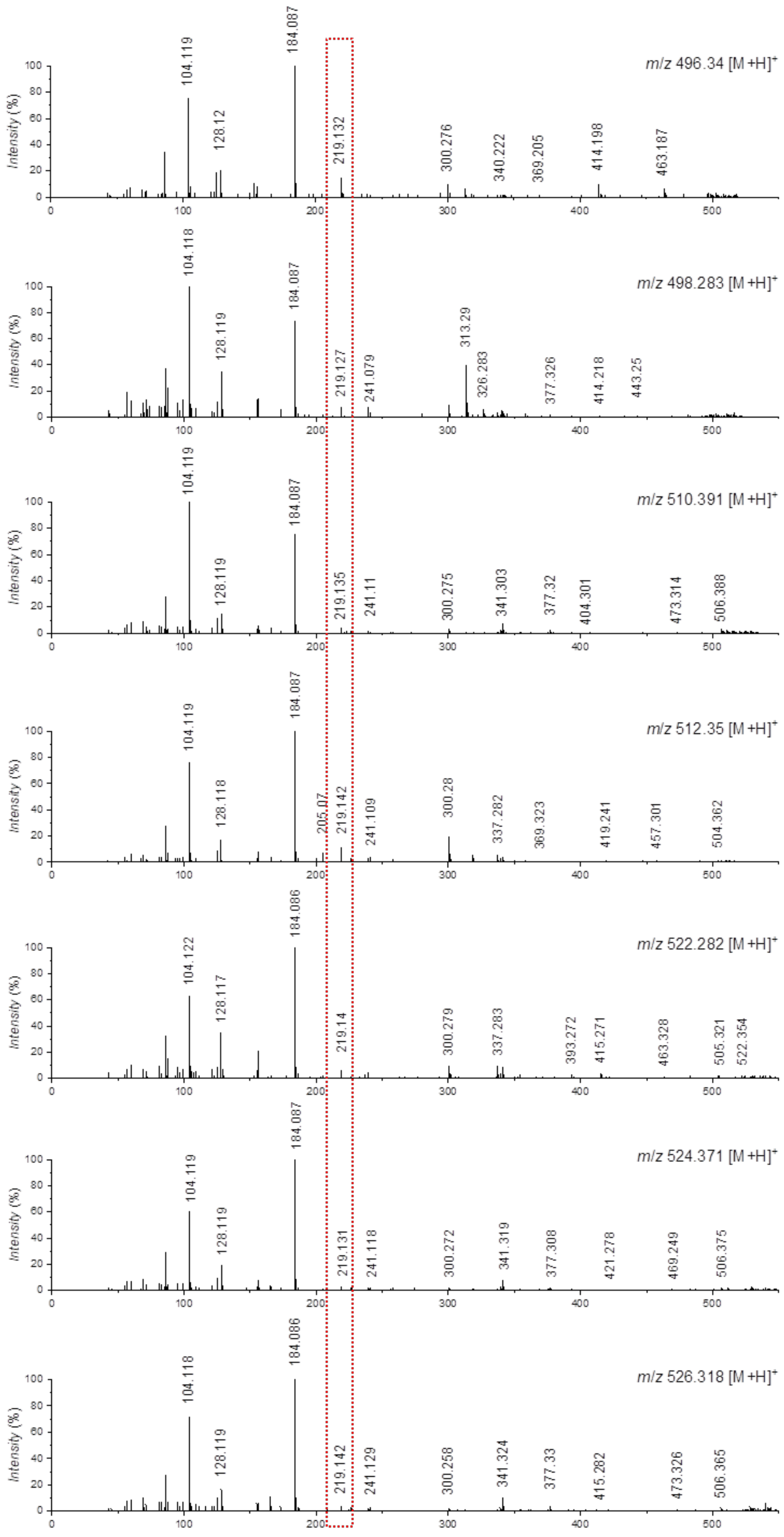

Figure S2. Cyclictetrapeptides -cluster and the MS/MS spectrum of each node

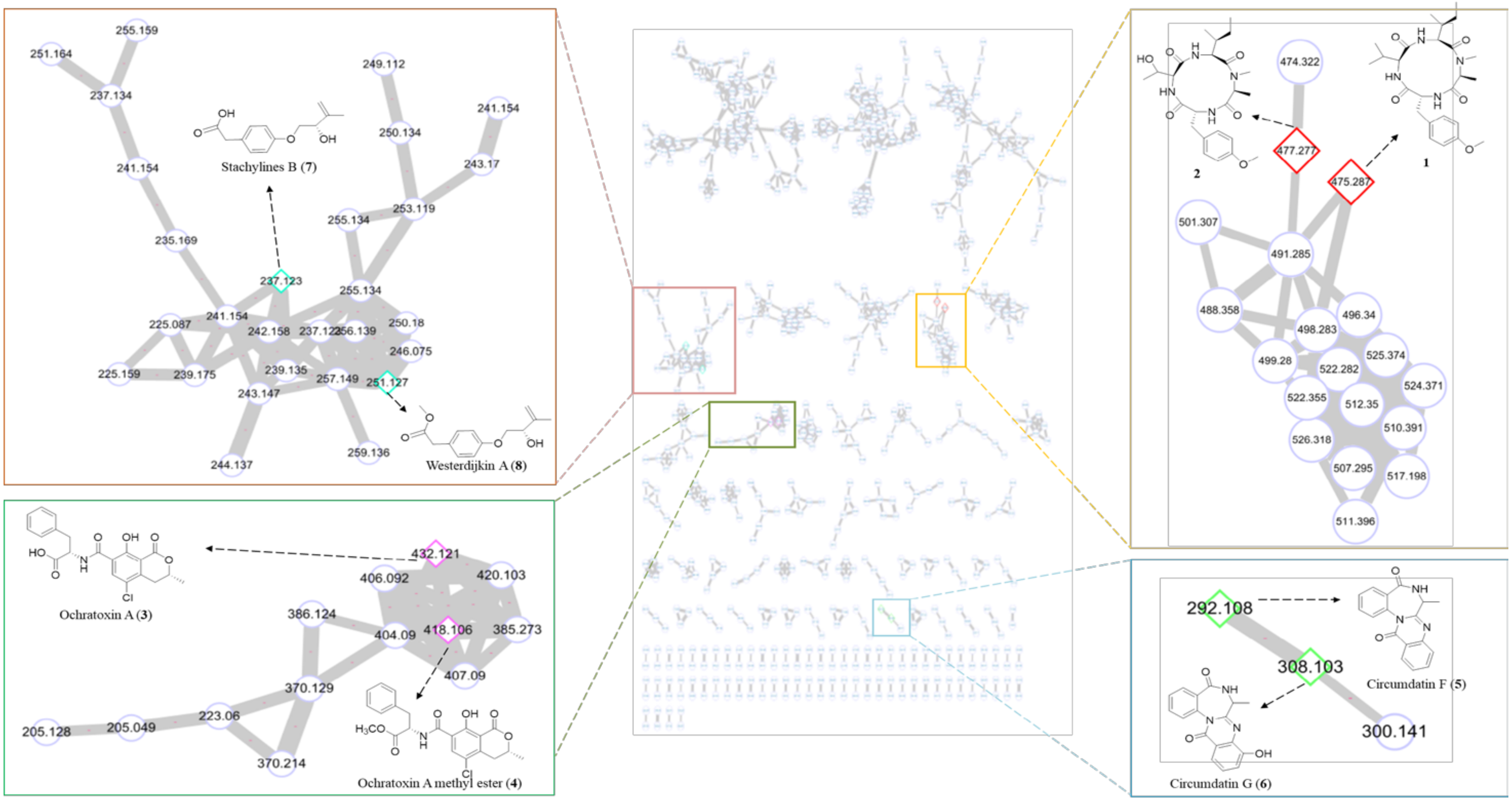

Figure S3. The cluster corresponding to compounds observed in the molecular networking

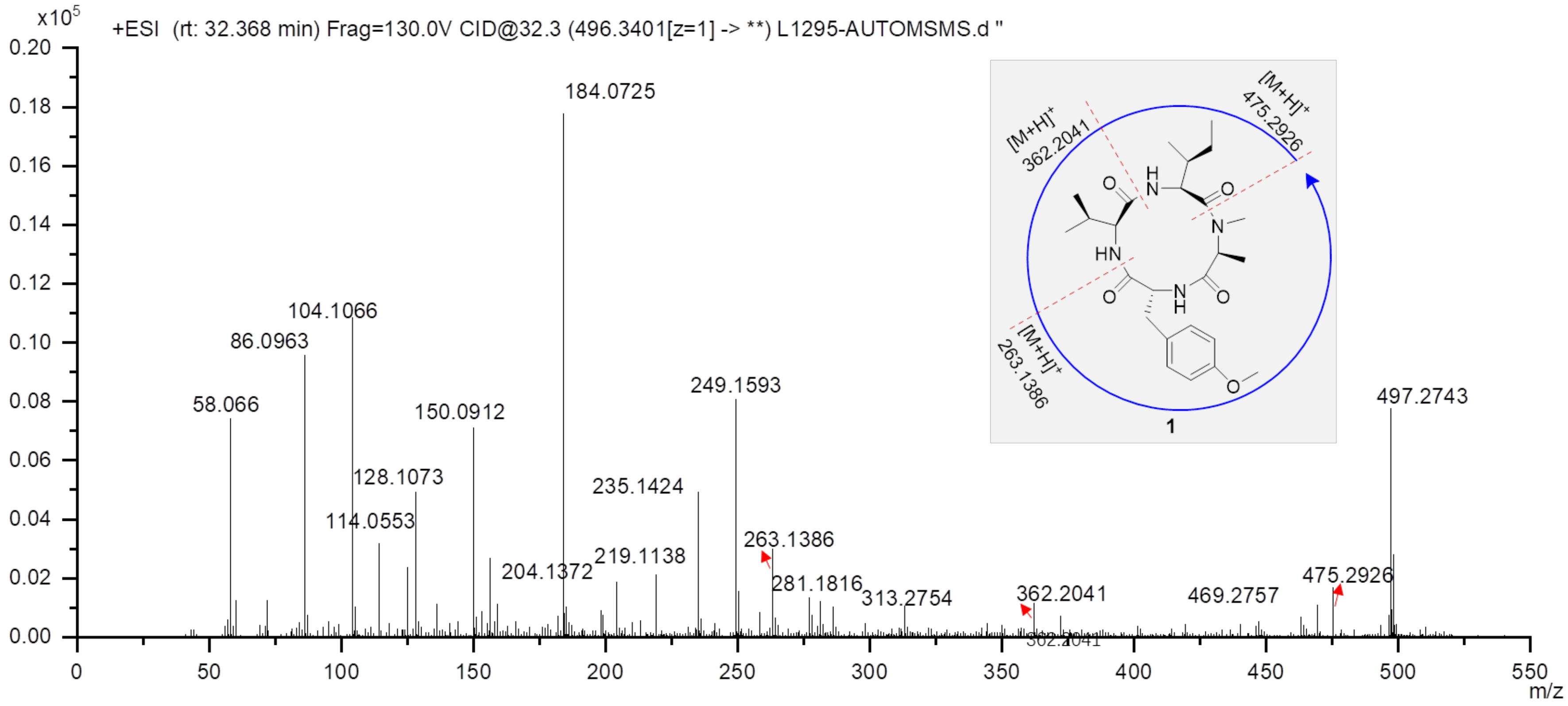

Figure S4. The ESI-MS/MS spectrum of **1.**

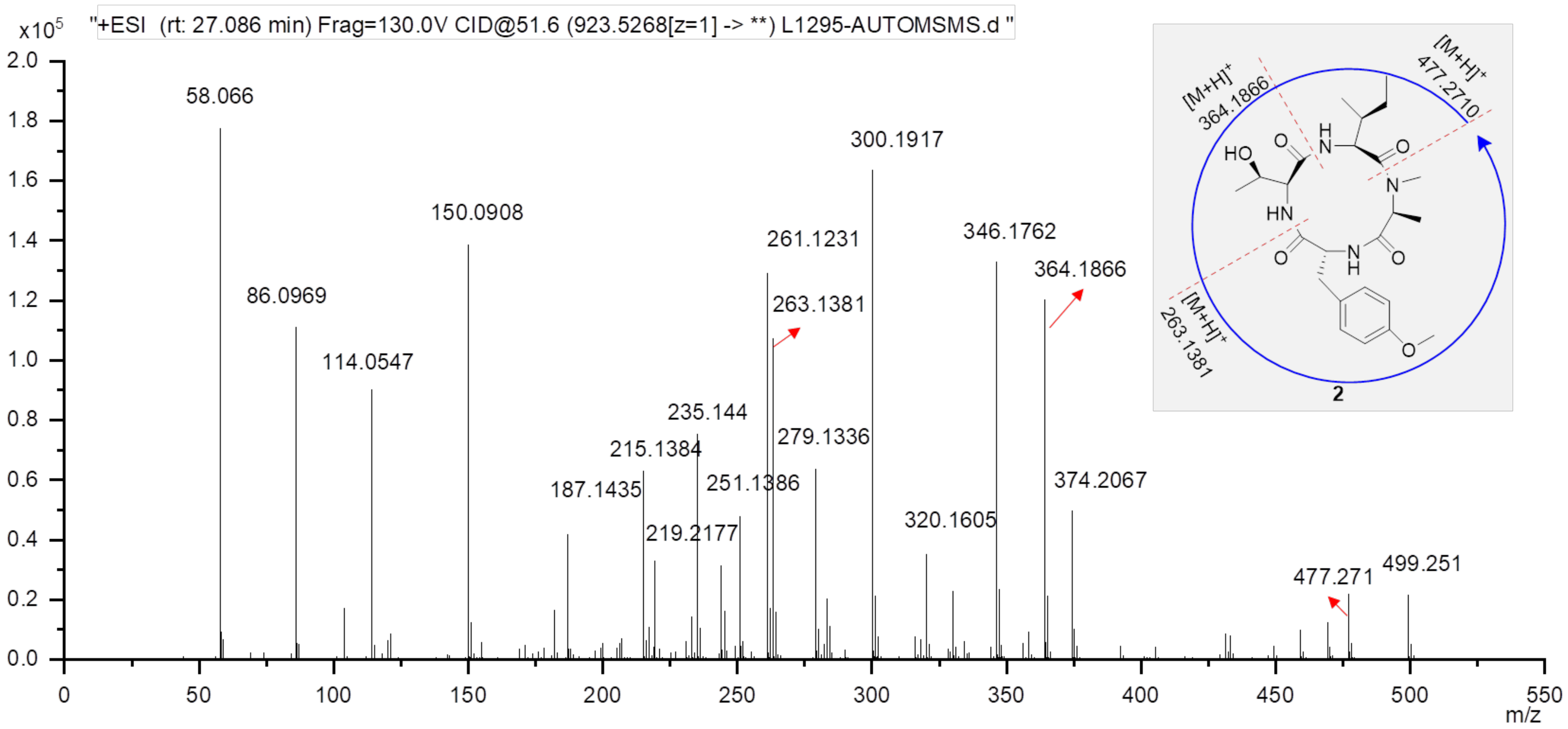

Figure S5. The ESI-MS/MS spectrum of **2.**

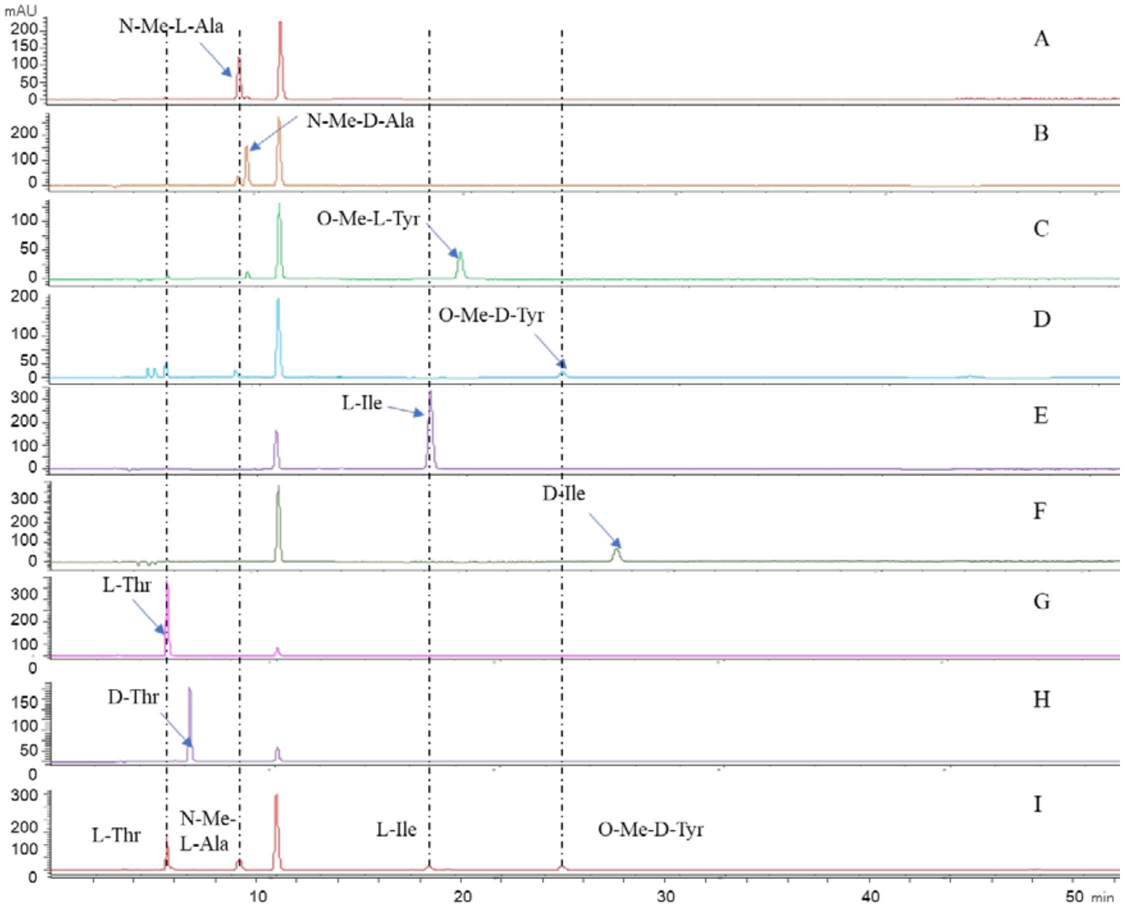

Figure S6. Advanced Marfey's analysis of compound **2.** (A-H): The retention times for the FDAA derivatives of *N*-Me-L-Ala, *N*-Me-D-Ala, *O*-Me-L-Tyr, *O*-Me-D-Tyr, L-Ile, D-Ile, L-Thr, and D-Thr, respectively. (I): The FDAA derivatives of the hydrolysate of **2**. The derivatives of the acid hydrolysate and the standard amino acids were subjected to RP HPLC analysis (Kromasil C18 column; 5 μm, 4.6 × 250mm; 1.0 mL/min; UV detection at 340 nm) with a linear gradient of acetonitrile (35%-45%) in water (TFA, 0.01%) over 40 min.

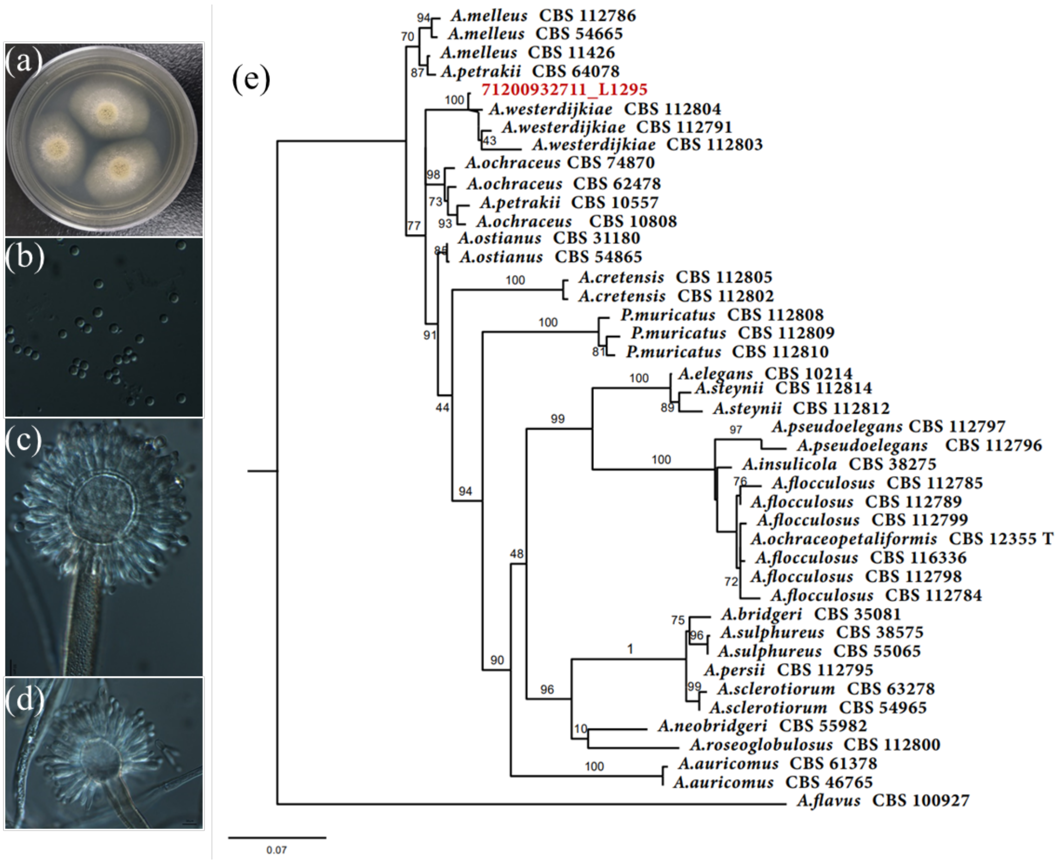

Figure S7. Phylogenetic analysis and morphological characters of *Aspergillus westerdijkiae* (a): Colonies on PDA after 7 d; (b): Conidiophores; (c-d): Conidia. Scale bars: b–d = 10 μm; (e). Phylogenetic analysis of *A. westerdijkiae* based on β-tubulin dataset.

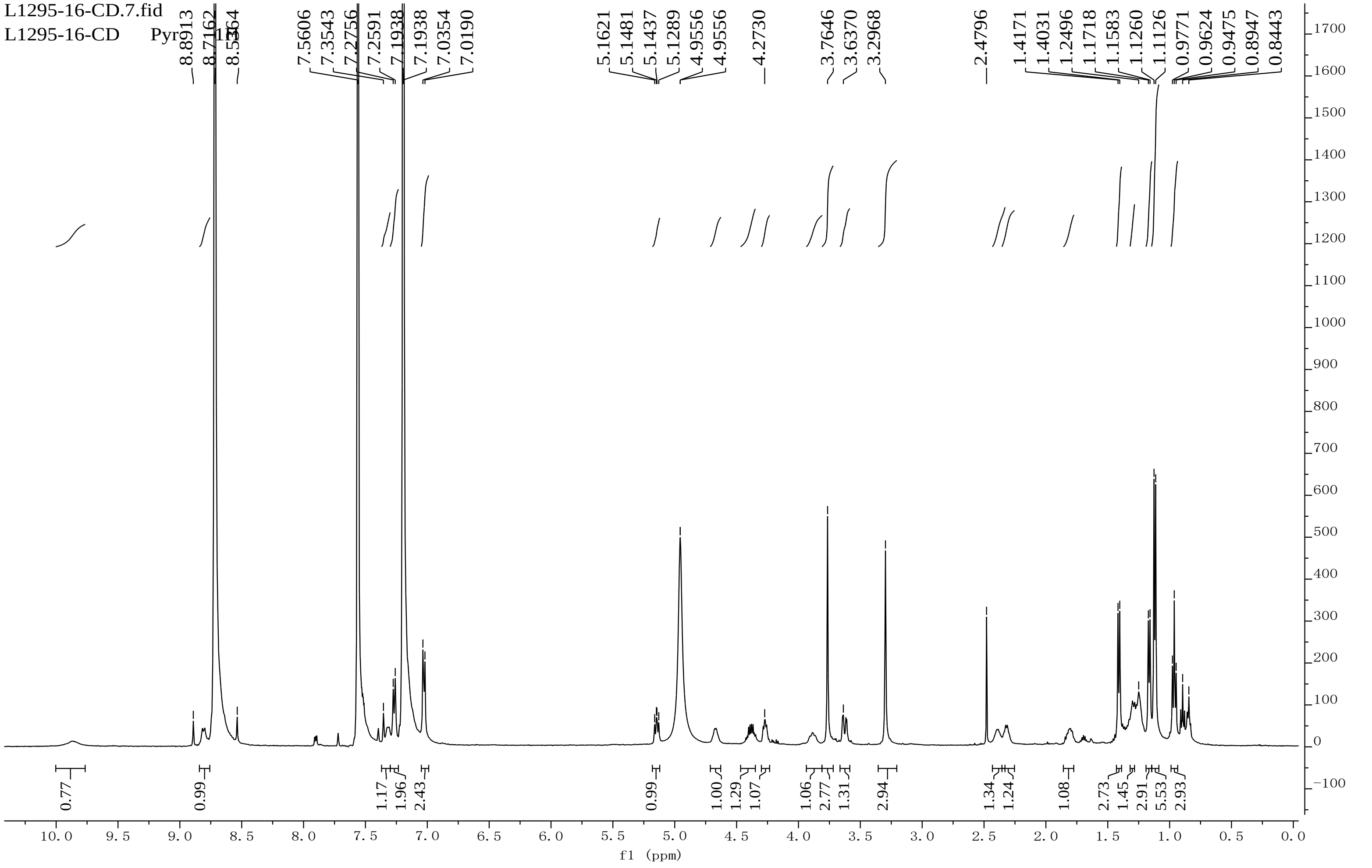

Figure S8. ^{1}H NMR spectrum of westertide A (**1**) in Pyridine-d_5 (500 MHz)

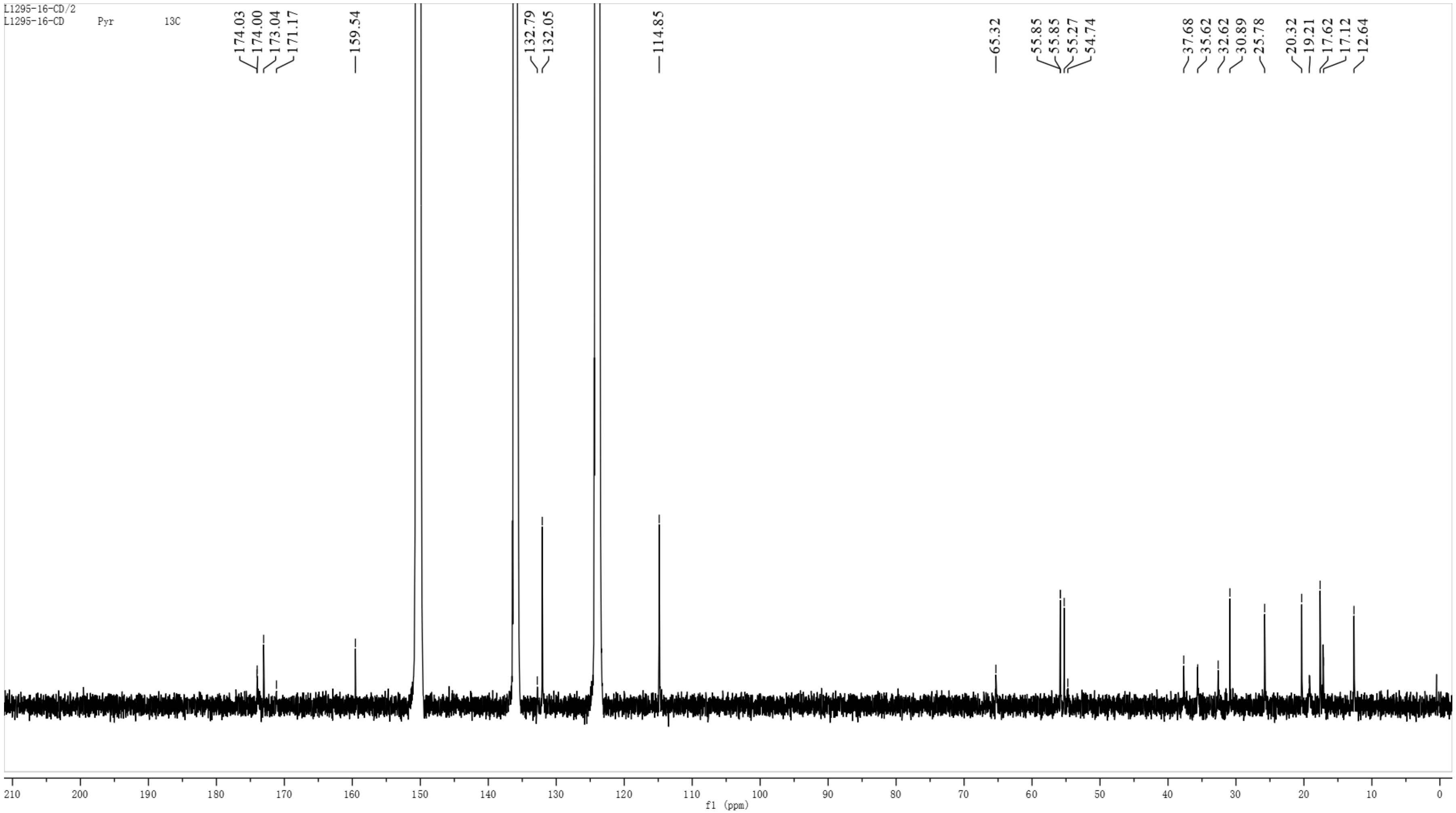

Figure S9. ^{13}C NMR spectrum of westertide A (**1**) in Pyridine-d_5 (125 MHz)

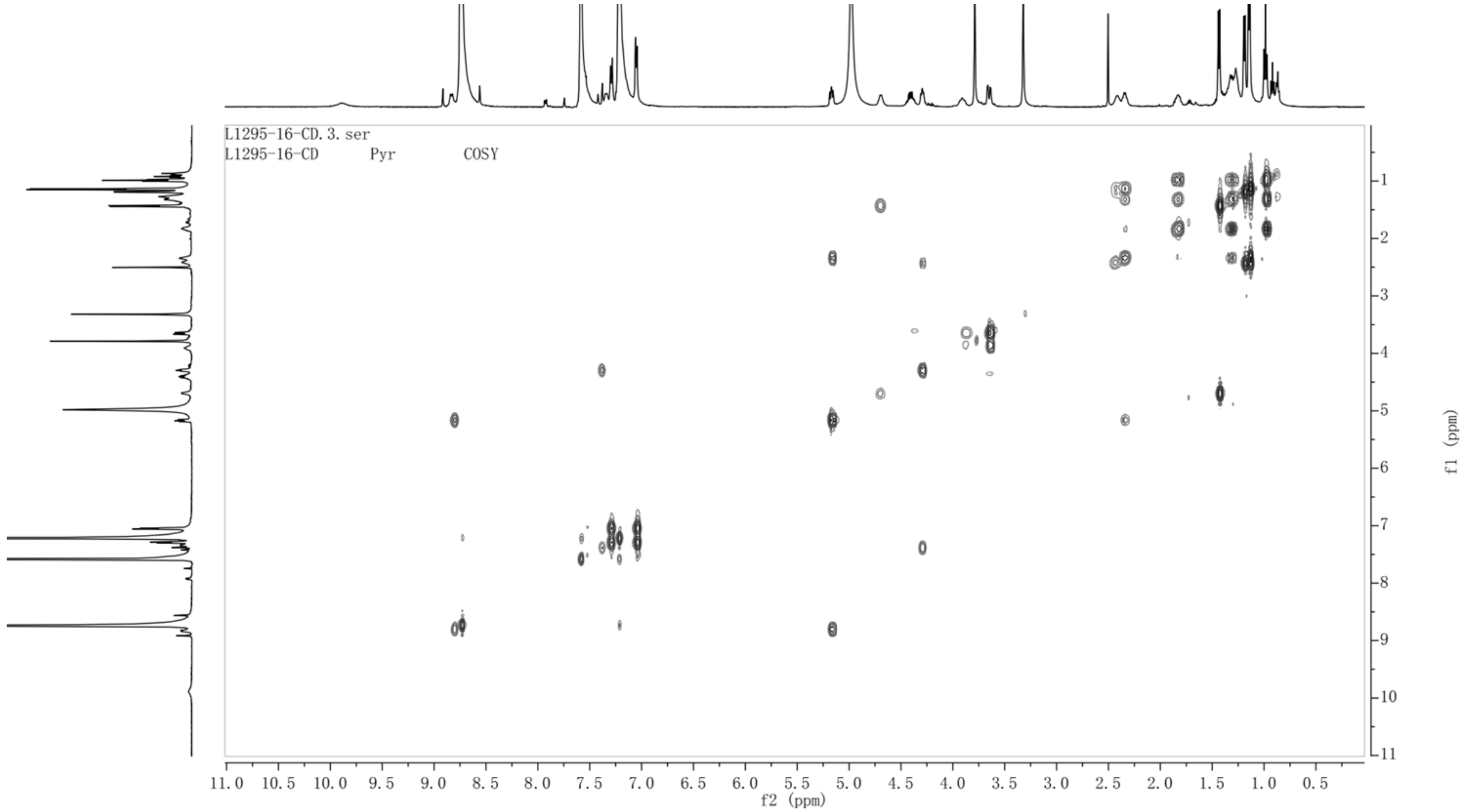

Figure S10. ^{1}H-^{1}H COSY spectrum of westertide A (**1**) in Pyridine-d_5

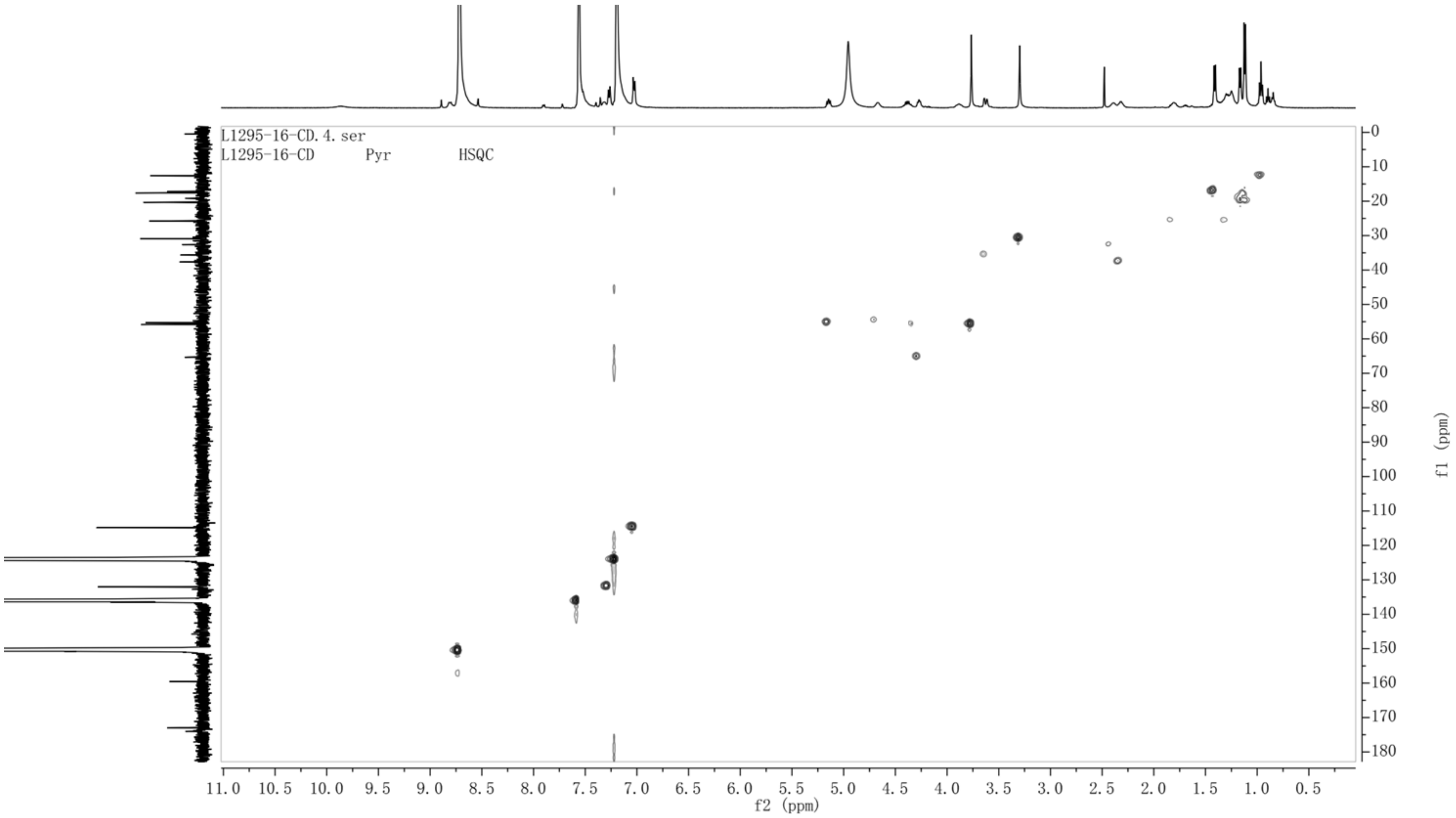

Figure S11. HSQC spectrum of westertide A (**1**) in Pyridine-d_5

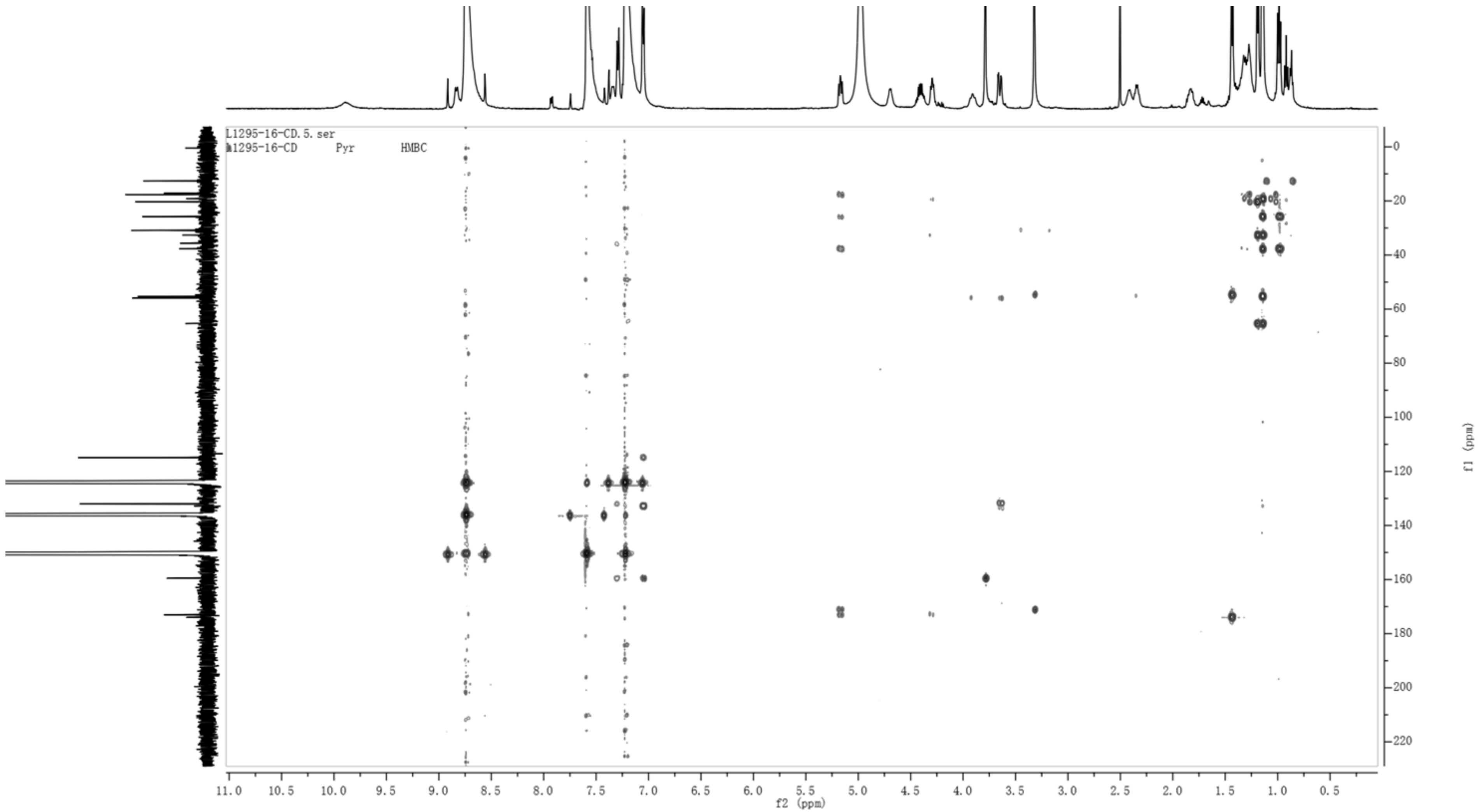

Figure S12. HMBC spectrum of westertide A (**1**) in Pyridine-d_5

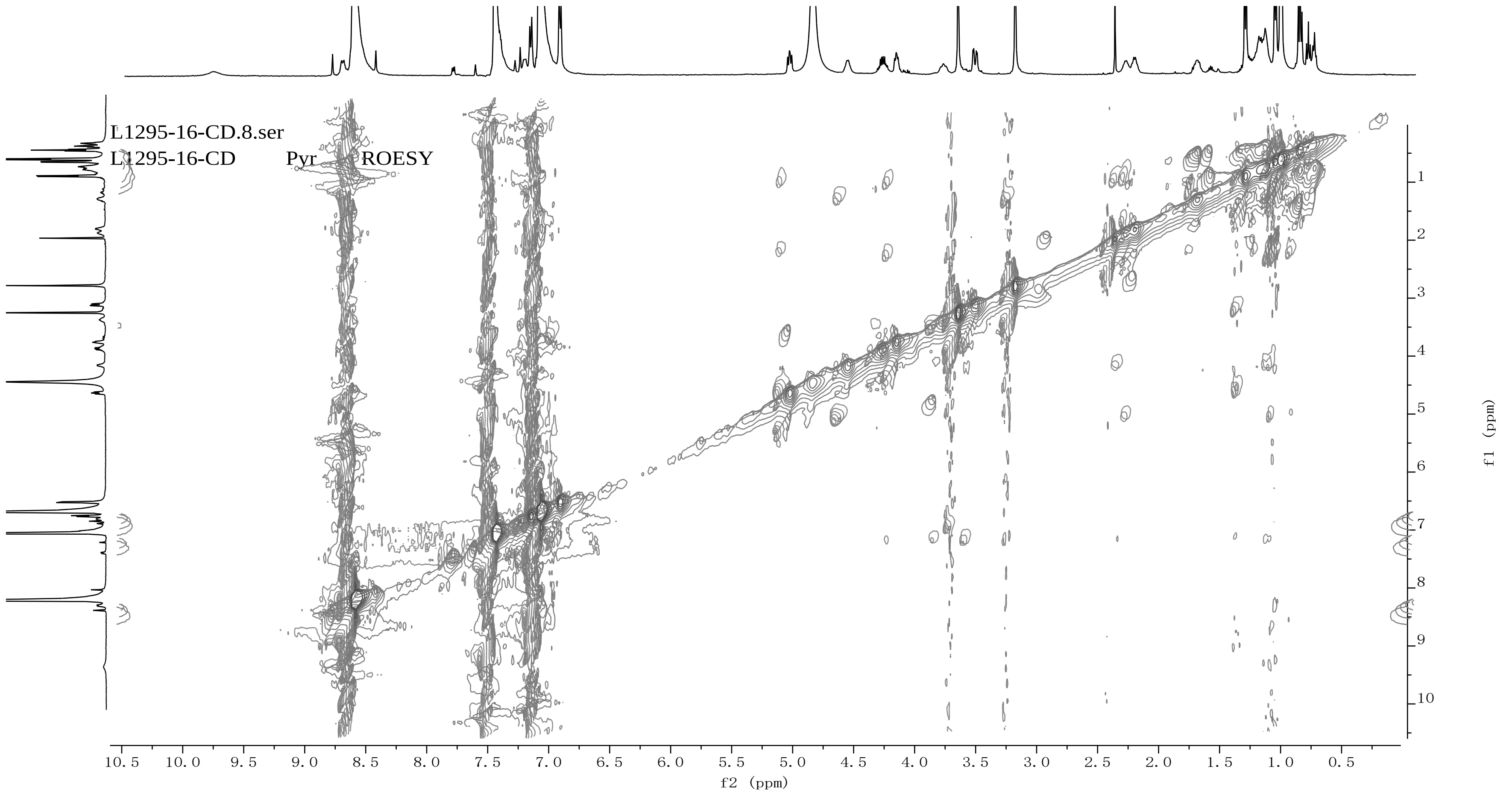

Figure S13. NOESY spectrum of westertide A (**1**) in Pyridine-d_5

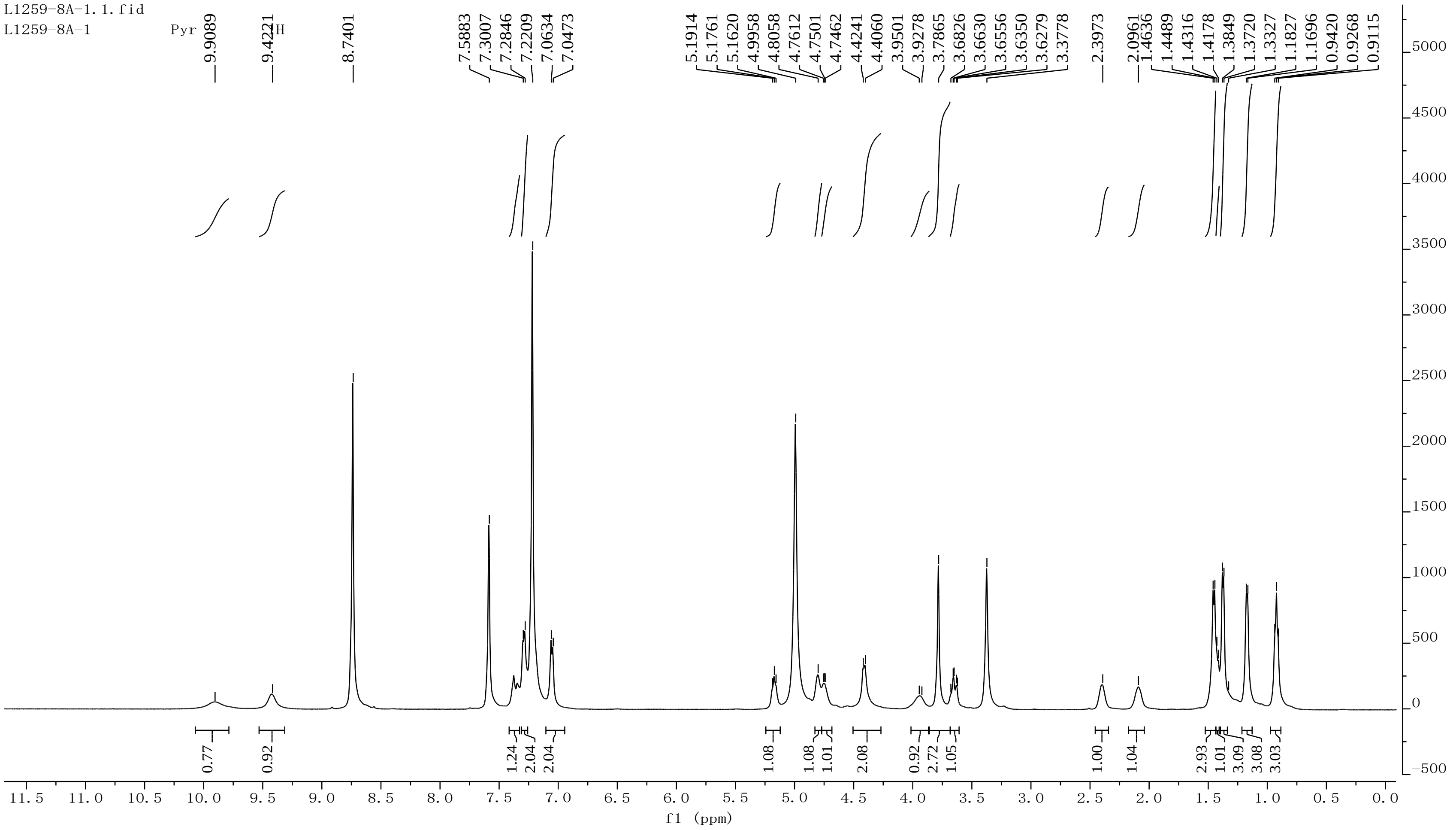

Figure S14. ^{1}H NMR spectrum of westertide B (**2**) in Pyridine-d_5 (500 MHz)

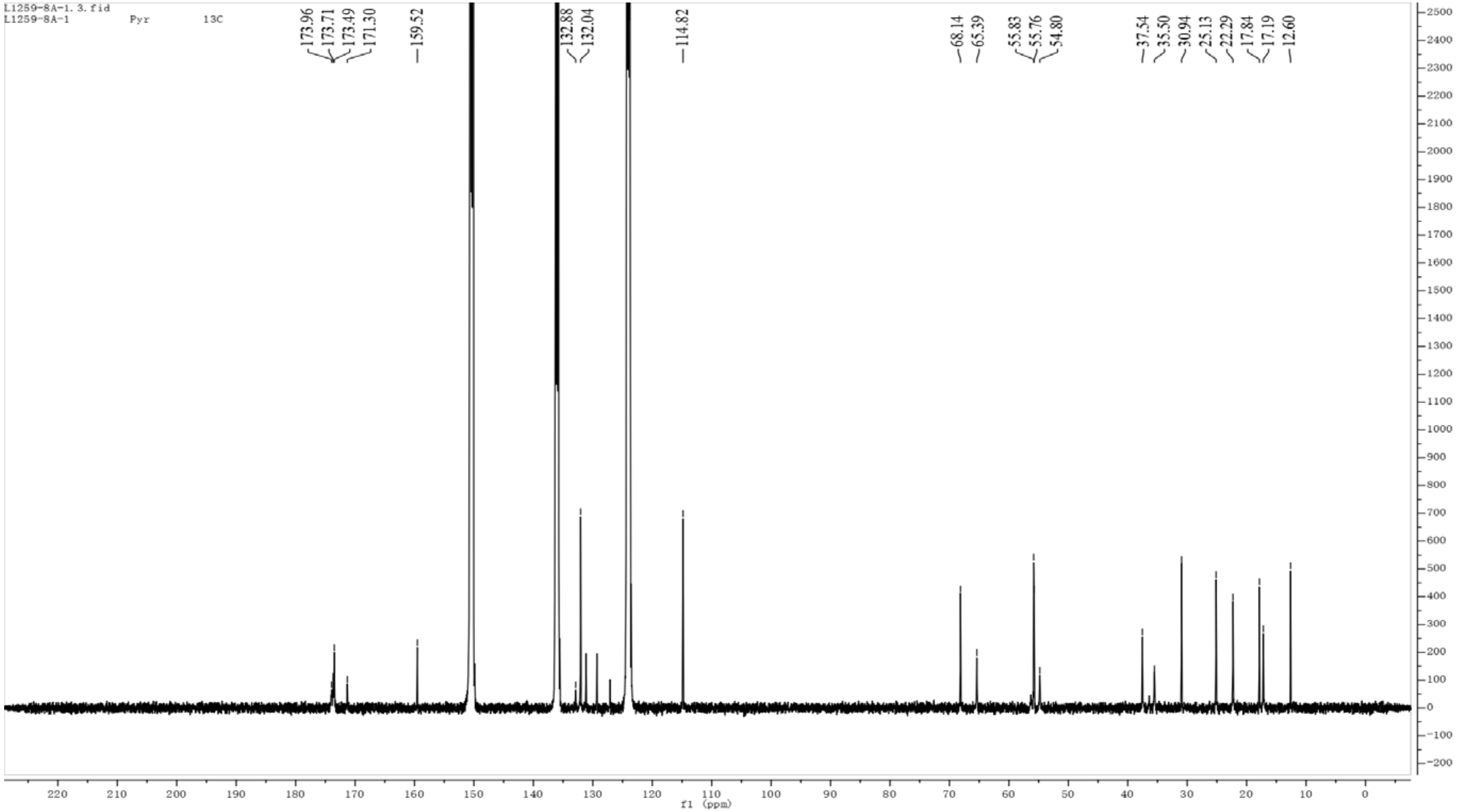

Figure S15. ^{13}C NMR spectrum of westertide B (**2**) in Pyridine-d_5 (125 MHz)

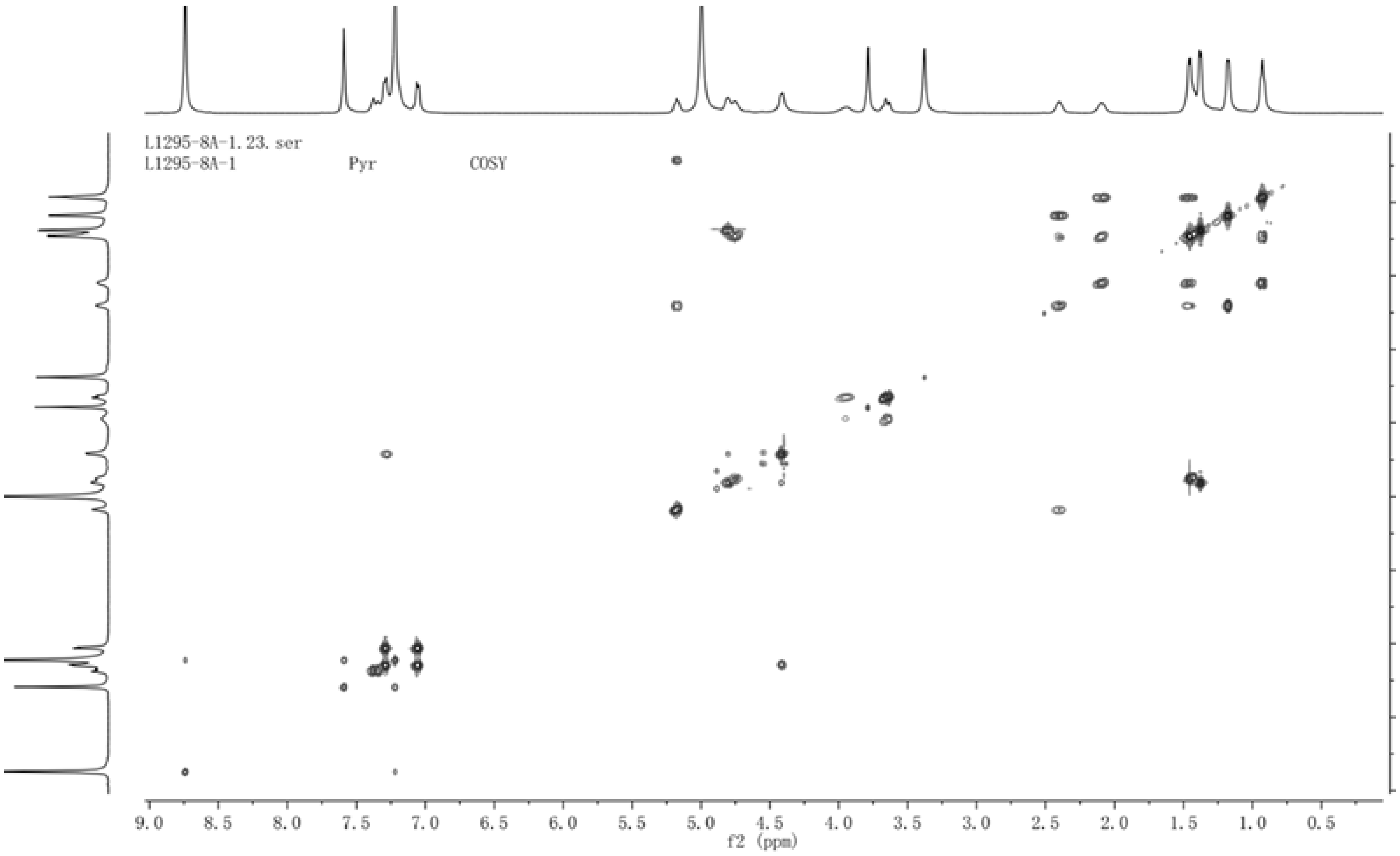

Figure S16. ^1H-^1H COSY spectrum of westertide B (**2**) in Pyridine-d_5

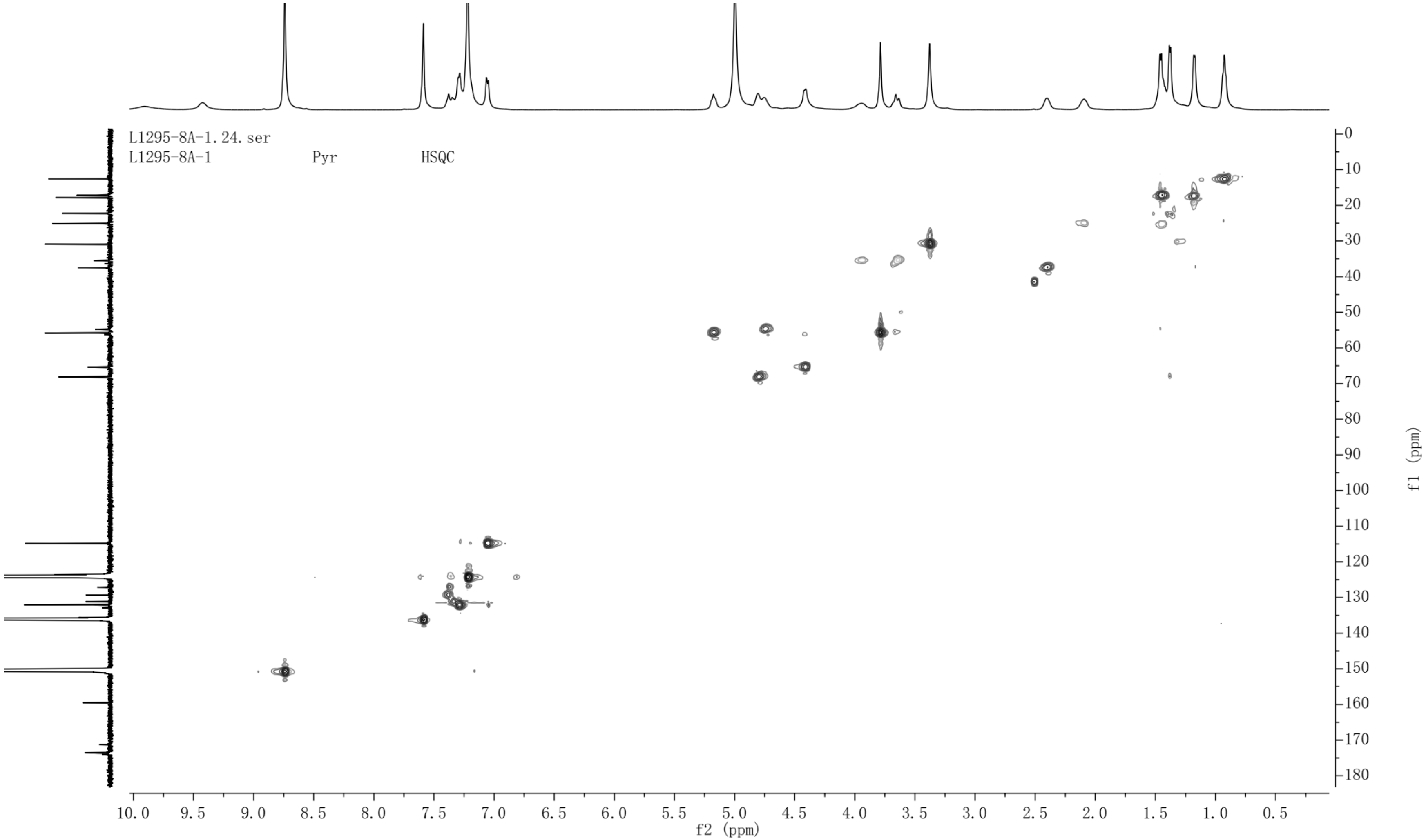

Figure S17. HSQC spectrum of westertide B (**2**) in Pyridine-d_5

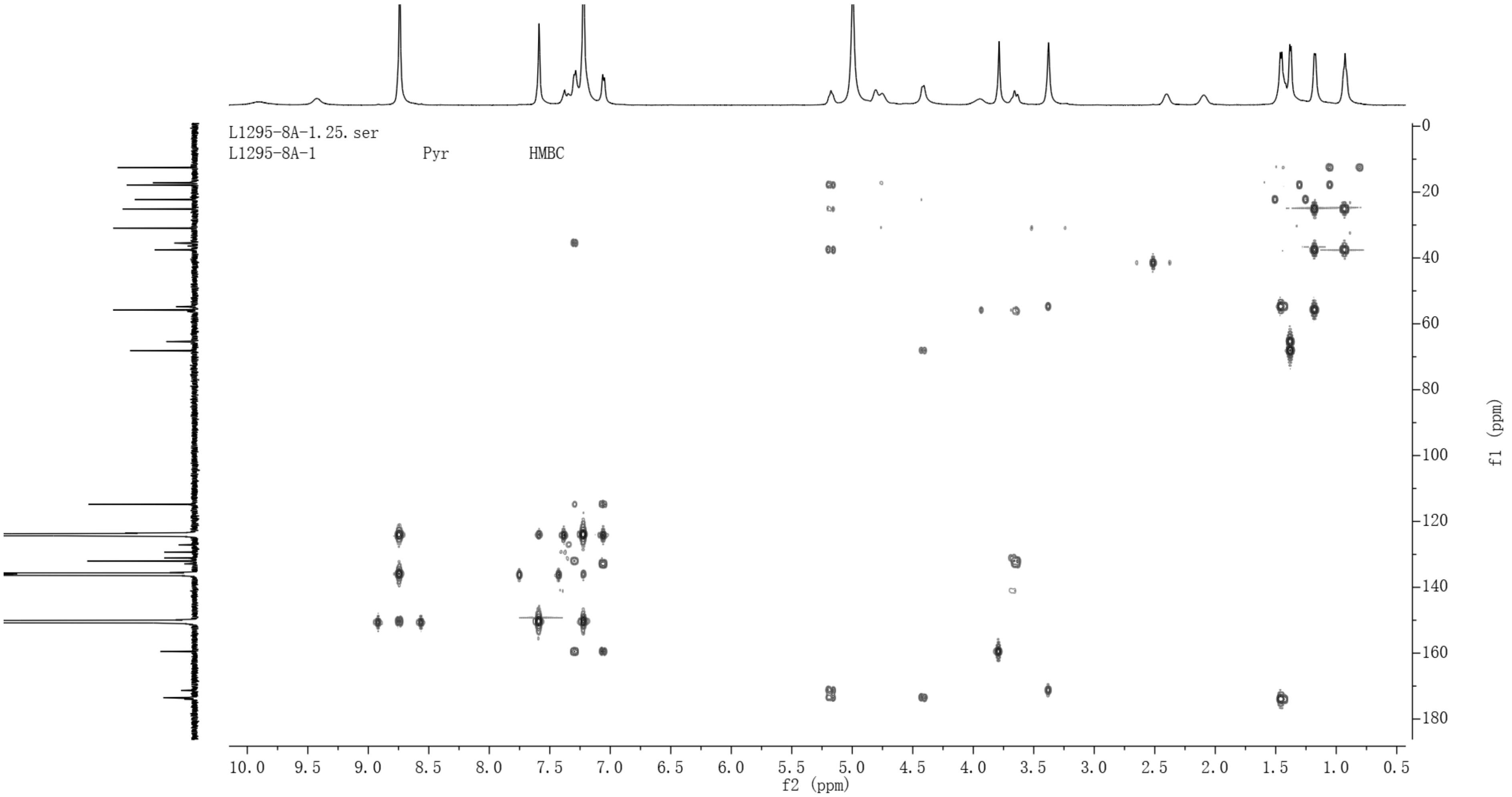

Figure S18. HMBC spectrum of westertide B (**2**) in Pyridine-d_5

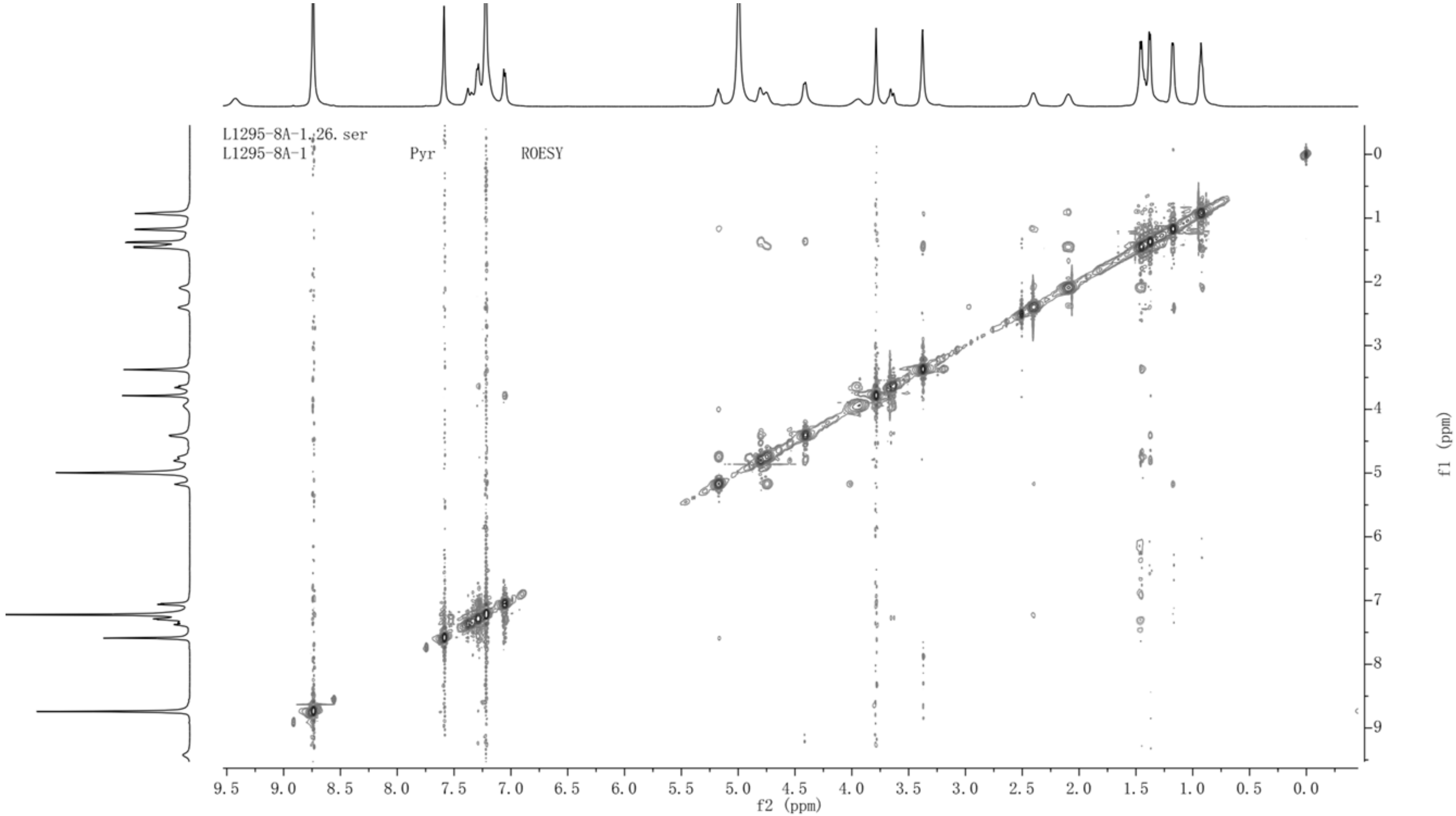

Figure S19. NOESY spectrum of westertide B (**2**) in Pyridine-d_5
